# Lack of GDAP1 Induces Neuronal Calcium and Mitochondrial Defects in a Knockout Mouse Model of Charcot-Marie-Tooth Neuropathy

**DOI:** 10.1371/journal.pgen.1005115

**Published:** 2015-04-10

**Authors:** Manuela Barneo-Muñoz, Paula Juárez, Azahara Civera-Tregón, Laura Yndriago, David Pla-Martin, Jennifer Zenker, Carmen Cuevas-Martín, Anna Estela, María Sánchez-Aragó, Jerónimo Forteza-Vila, José M. Cuezva, Roman Chrast, Francesc Palau

**Affiliations:** 1 Program in Rare and Genetic Diseases and IBV/CSIC Associated Unit, Centro de Investigación Príncipe Felipe, Valencia, Spain; 2 CIBER de Enfermedades Raras (CIBERER), ISCIII, Valencia and Madrid, Spain; 3 Department of Medical Genetics, University of Lausanne, Lausanne, Switzerland; 4 Centro de Biología Molecular Severo Ochoa, UAM-CSIC, Madrid, Spain; 5 Instituto de Investigación Hospital 12 de Octubre, Universidad Autónoma de Madrid, Madrid, Spain; 6 Instituto Valenciano de Patología, Catholic University of Valencia, Valencia, Spain; 7 Department of Neuroscience and Department of Clinical Neuroscience, Karolinska Institutet, Stockholm, Sweden; 8 University of Castilla-La Mancha School of Medicine at Ciudad Real, Ciudad Real, Spain; The Jackson Laboratory, UNITED STATES

## Abstract

Mutations in *GDAP1*, which encodes protein located in the mitochondrial outer membrane, cause axonal recessive (AR-CMT2), axonal dominant (CMT2K) and demyelinating recessive (CMT4A) forms of Charcot-Marie-Tooth (CMT) neuropathy. Loss of function recessive mutations in *GDAP1* are associated with decreased mitochondrial fission activity, while dominant mutations result in impairment of mitochondrial fusion with increased production of reactive oxygen species and susceptibility to apoptotic stimuli. *GDAP1* silencing *in vitro* reduces Ca^2+^ inflow through store-operated Ca^2+^ entry (SOCE) upon mobilization of endoplasmic reticulum (ER) Ca^2+^, likely in association with an abnormal distribution of the mitochondrial network. To investigate the functional consequences of lack of GDAP1 *in vivo*, we generated a *Gdap1* knockout mouse. The affected animals presented abnormal motor behavior starting at the age of 3 months. Electrophysiological and biochemical studies confirmed the axonal nature of the neuropathy whereas histopathological studies over time showed progressive loss of motor neurons (MNs) in the anterior horn of the spinal cord and defects in neuromuscular junctions. Analyses of cultured embryonic MNs and adult dorsal root ganglia neurons from affected animals demonstrated large and defective mitochondria, changes in the ER cisternae, reduced acetylation of cytoskeletal α-tubulin and increased autophagy vesicles. Importantly, MNs showed reduced cytosolic calcium and SOCE response. The development and characterization of the *GDAP1* neuropathy mice model thus revealed that some of the pathophysiological changes present in axonal recessive form of the *GDAP1*-related CMT might be the consequence of changes in the mitochondrial network biology and mitochondria–endoplasmic reticulum interaction leading to abnormalities in calcium homeostasis.

## Author Summary

Charcot-Marie-Tooth (CMT) disease is an inherited motor and sensory peripheral neuropathy. Mutations in the *GDAP1* gene cause either an axonapathy or an myelinopathy that can be transmitted recessively or dominantly to offspring. GDAP1 is located in the mitochondrial outer membrane and seems to participate in the mitochondrial network dynamics. To investigate the biological and functional consequences of lack of GDAP1 and to gain insight into the pathophysiology of the *GDAP1*-related neuropathies we have generated a *Gdap1* knockout mouse. Characterization of this model revealed that the absence of GDAP1 induces a peripheral neuropathy with loss of motor neurons and abnormal neuromuscular junctions. We also observed defects in embryonic motor neurons and adult dorsal root ganglia sensory neurons derived from affected animals. Specifically, cultured motor neurons showed large and abnormal mitochondria, dilated perinuclear space and endoplasmic reticulum, changes in acetylation of cytoskeletal α-tubulin and calcium depletion. We propose that pathophysiology of *GDAP1*-associated recessive CMT neuropathy may be the consequence of abnormal calcium homeostasis and changes in the mitochondrial network biology and mitochondria–endoplasmic reticulum interactions. Our findings may be also relevant to understand the role of GDAP1 in relation to other neuropathy-related mitochondrial proteins such as mitofusin 2.

## Introduction

Charcot-Marie-Tooth (CMT) disease is one of the most frequent inherited neurological disorders and is characterized by either demyelinating or axonal neuropathy of motor and sensory peripheral nerves [1–3]. More than eighty genes are related to the pathogenesis of CMT and other peripheral neuropathies affecting a wide number of biological functions and cellular pathways [4,5] (http://neuromuscular.wustl.edu/time/hmsn.html). Mutations in *GDAP1*, which maps at human chromosome 8q21.1, are causative for several types of neuropathy and are transmitted through various modes of inheritance including autosomal recessive demyelinating CMT4A with reduced nerve condition velocities (NCVs) [6], autosomal recessive axonal AR-CMT2K with preserved NCVs and abnormal compound motor action potentials (CMAPs) [7], and the less frequent autosomal dominant CMT2K [8,9] and recessive intermediate RI-CMT [10].

GDAP1 is a protein of 358 amino acids located at the mitochondrial outer membrane (MOM) [11,12] and has two glutation S-transferase (GST) domains, with a protein interacting α4-α5 loop domain between the two GST domains, and one transmembrane C-terminal domain that is important for the correct anchoring of the protein to the MOM [13]. GDAP1 is mainly expressed in neurons [14] but expression in Schwann cells has also been reported [11]. GDAP1 plays a role in the regulation of mitochondrial dynamics inducing organelle fission [11,12]. Whereas loss of function recessive mutations are associated with decreased fission activity, dominant mutations result in impairment of mitochondrial fusion with increased production of reactive oxygen species (ROS) and susceptibility to apoptotic stimuli [15]. Through characterization of fibroblasts derived from CMT4A patients, Noack and colleagues [16] observed that GDAP1 is implicated in the control of the cellular glutathione content and mitochondrial activity, supporting the role of GDAP1 in the defense against oxidative stress. We have recently demonstrated that *GDAP1* silencing in the neuroblastoma SH-SY5Y cells reduces Ca^2+^ inflow through store-operated Ca^2+^ entry (SOCE) upon mobilization of endoplasmic reticulum (ER) Ca^2+^, likely in association with the abnormal distribution of the mitochondrial network [17]. Thus, the pathophysiology of *GDAP1*-related neuropathies may involve complex cellular interactions between mitochondrial network and ER, oxidative stress and Ca^2+^ homeostasis, associated with mitochondrial dynamics and distribution.

Both demyelinating [6,18] and axonal [7,19] lesions have been reported in the nerve biopsy from *GDAP1*-related CMT patients. However, electrophysiological studies indicate that especially in the more severe patients the *GDAP1* mutations induce primary axonal neuropathy with secondary demyelination [19,20]. Data of mitochondrial morphology in biopsy specimens are scarce, but in those cases when biopsy data were presented no abnormalities were reported [1,21].

To investigate the functional consequences of loss of GDAP1 function *in vivo*, we generated a *Gdap1* knockout mouse *(Gdap1* ^*-/-*^ *)*. Here, we describe characterization of this model and report that the *Gdap1* ^-/-^ mice display neuropathic behavior and symptoms closely similar to those of AR-CMT2K patients. Detailed characterization of the generated *in vivo* model allowed us to propose a pathogenic mechanism of the disease that involves depletion of cellular calcium associated with changes in the mitochondrial network.

## Results

### Generation of *Gdap1* ^-/-^ mice

**Fig 1:**
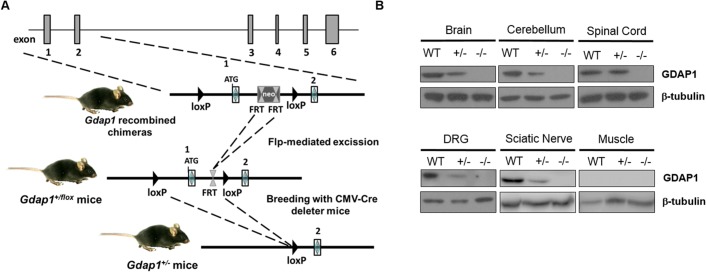
Generation of *Gdap1* ^*-/-*^ mice. **(A)** Schematic representation of *Gdap1* ^*-/-*^ targeting strategy. Diagram is not to scale. Hatched rectangles represent *Gdap1* exons 1 to 6, solid line represents mouse chromosome 1. FRT sites are represented by double triangles and *lox*P sites are right-faced triangles. **(B)** GDAP1 protein expression was assessed by immunoblotting of selected tissue homogenates prepared from 2 months-old wild-type (WT), *Gdap1* ^*+/-*^ (+/-) and *Gdap1* ^-/-^ (-/-) mice.

To generate the *Gdap1* knockout mice (*Gdap1* ^-/-^) we deleted exon 1 of *Gdap1* by homologous recombination. This was achieved by using a targeting vector containing a self-excising lox*P*-exon 1 *Gdap1*-FRT-PGK-Neo^R^-FRT-lox*P* cassette (Fig 1A), which allows to generate a recombined locus by deletion of the selectable marker Neo. The targeting construct was electroporated into 129sv stem cells and 2 recombinant clones were identified by both PCR and Southern blotting. *Gdap1* ^-/-^ mice were established by breeding heterozygous *Gdap1* ^*+/flox*^ mice with *CMV-Cre* deleter mice leading to ubiquitous exon 1 deletion. *Gdap1* ^-/-^ mice are born at expected Mendelian ratios, have normal life-span and are fertile. Western blot analysis of neuronal and non-neuronal tissue lysates of *Gdap1* ^*+/+*^ (WT), *Gdap1* ^*+/-*^ and *Gdap1* ^-/-^ littermates mice confirmed the absence of GDAP1 protein (Fig 1B).

### *Gdap1* ^-/-^ mice develop motor deficits

**Fig 2:**
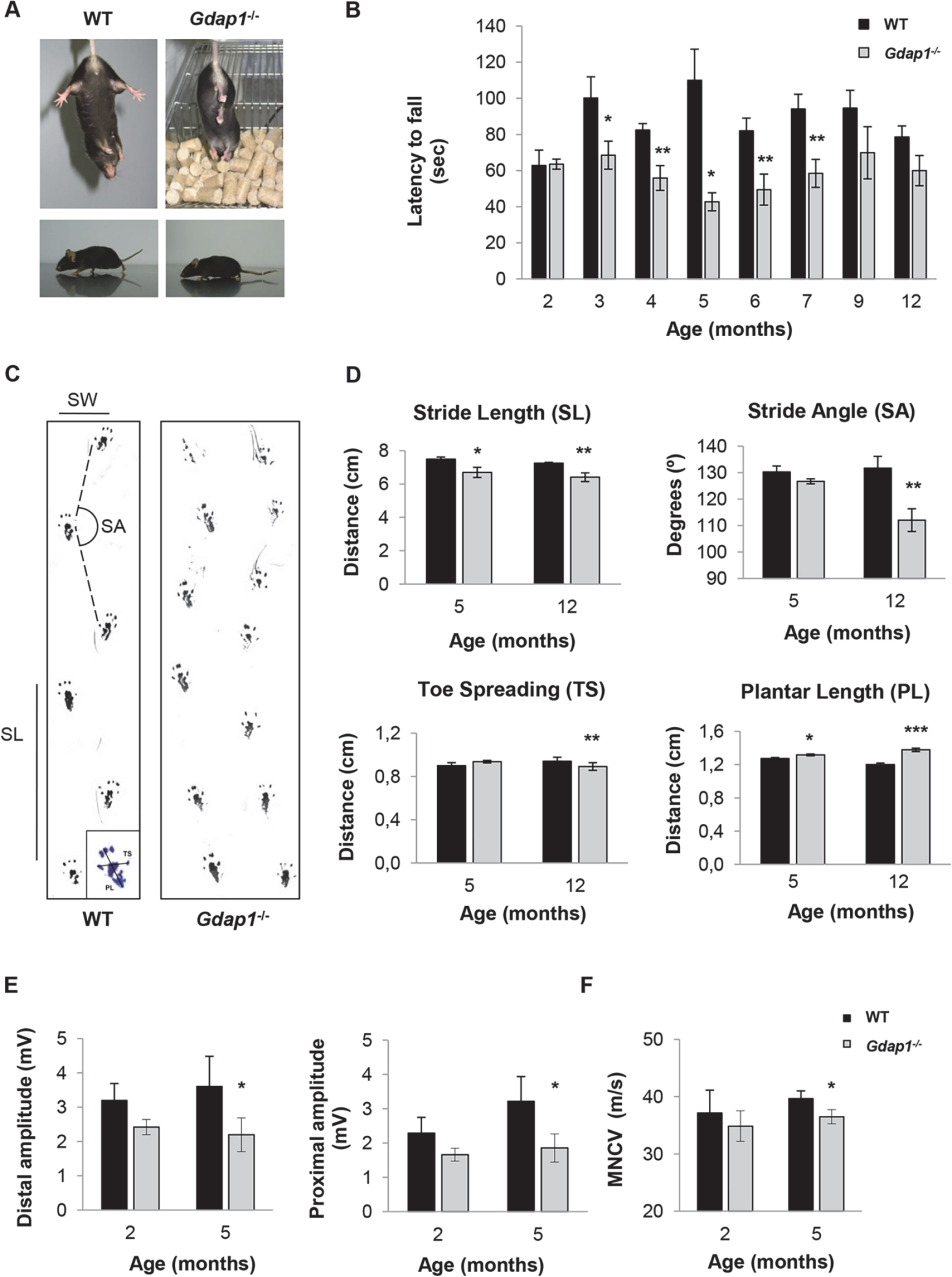
Behavioural testing and electrophysiological measurements on *Gdap1* ^*-/-*^ mice. **(A)** Upper panel shows photographs of 3 months-old mice suspended by its tail. WT mice show a characteristic response trying to escape by splaying its hind limbs away from the trunk of its body. In contrast, hind limbs of *Gdap1* ^-/-^ mice are held tonically against its trunk in an abnormal dystonic posture. Lower panels display a low body position and a dragging tail present in *Gdap1* ^*-/-*^ mice as compared to age-matched WT mice. **(B)** Motor coordination was assessed by rotarod test, (n = 10 for each genotype and at each age group). **(C)** Representative hind limb walking patterns of 5 months-old WT and *Gdap1* ^-/-^ mice where the stride length (SL) and stride angle (SA) have been depicted. Footprints revealed that *Gdap1* ^*-/-*^ mice walk with an abnormal gait. The scheme of a hindpaw footprint indicating measured parameters (PL: plantar length; TS: toe spreading) has been included. **(D)** Quantification of various parameters obtained from the gait analysis of WT (black columns) and *Gdap1* ^*-/-*^ (grey columns) animals at 5 and 12 months of age. Upper graphs show stride length (left) and stride angle (right). Lower graphs show the quantitative analysis of the hindpaw footprint parameters toe spreading (left) and plantar length (right). Analysis was conducted on 10 clearly visible footprints at 5 animals per genotype. Determination of sciatic nerve compound muscle action potential (CMAP) amplitudes at both distal and proximal **(E)** as well as motor nerve conduction velocities (MNCV) **(F)** measured in WT and *Gdap1* ^-/-^ mice at 2 and 5 months of age (n = 4). Error bars indicate standard error of the mean (S.E.M.). *p* values were calculated using Student's *t* test,*p<0.05, **p<0.001, ***p<0.0001.

The severe recessive form of *GDAP1*-related CMT starts early in infancy or childhood with weakness and wasting of the feet followed by involvement of the hands leading to pronounced disability. Patients are usually wheelchair bound starting from the second decade of life. Thus, we compared motor behavior between *Gdap1* ^-/-^ and WT mice at the early adult age. We observed abnormal hind-limb clasping reflex in *Gdap1* ^-/-^ mice at the age of 3 months (Fig 2A, upper panels). When observed during locomotion, most animals showed very low position of the body and dragging tail indicating presence of motor deficits (Fig 2A, lower panels). Then we tested motor and coordination behavior by rotarod test in several ages. In order to avoid previous learning we used different animals for each time point. We detected significantly reduced latency to fall in 3 months-old mutant mice. The persistence of this motor behavior was confirmed in older *Gdap1* ^-/-^ mice from 4 to 7 months of age but became more variable and thus non-significant after 9 months of age (Fig 2B).

In order to further characterize motor phenotype of *Gdap1* ^-/-^ mice we analyzed their gait behavior at the age of 5 and 12 months (Fig 2C). At both time-points *Gdap1* ^-/-^ mice had shorter stride length (p<0.05 at 5 months and p<0.01 at 12 months, Student’s *t* test) and at 12 months we also detected more narrow gait angles (p<0.001, Student’s *t* test) (Fig 2D, upper panels). As we observed everted paws in some mutant animals, we also analyzed the plantar print (Fig 2D, lower panels). At the age of 12 months we detected significant decrease of toe spreading and an increased extension of plantar length that suggest a defect on the plantar muscle strength. The observed defects in motor behavior in mice lacking GDAP1 prompted us to further investigate their electrophysiological properties along the sciatic nerve. Therefore, we measured compound muscle action potential (CMAP) amplitude (Fig 2E) and the motor nerve conduction velocity (MNCV) (Fig 2F). In line with the absence of detectable behavioral phenotype, we were not able to detect any electrophysiological differences between *Gdap1* ^-/-^ and WT mice at 2-months of age. Interestingly, at 5-months *Gdap1* ^-/-^ mice showed a significant reduction of CMAP amplitude obtained for distal (at the ankle) and proximal (at the hip) stimulation, indicative for an axonal neuropathy (Fig 2E, p<0.05, Student’s *t* test). Concomitantly, at this age, we observed a slight but significant reduction in MNCV for *Gdap1* ^-/-^ mice as compared to WT mice (Fig 2F, p<0.05, Student’s *t* test). However, we did not measure CMAP and MNCV at later time points to assess the persistence of this effect.

Slight reductions in MNCV can be associated with axonal neuropathies [19]. In *GDAP1*-CMT patients, axonal neuropathy is associated with loss of axons in sural nerve biopsies [19,22]. We therefore decided to further investigate the nerve morphology of sciatic nerves from 5-months-old *Gdap1* ^-/-^ and age-matched WT mice, the age at which we detected electrophysiological changes compatible with presence of axonopathy (Fig 2). Surprisingly, we did not detect any significant reduction in the axonal number correlating with the observed reduction in CMAP amplitude in 5 months-old mice (S1A and S1B Fig). Distribution of axon sizes in both proximal and distal sciatic nerves did not show any differences between *Gdap1* ^-/-^ and WT mice (S1C Fig). Moreover, myelin thickness was preserved in 5-months old *Gdap1* ^-/-^ mice as well. By measuring g-ratio (ratio of axon diameter versus fiber diameter) in proximal and distal sciatic nerves of both *Gdap1* ^*-/-*^ and WT mice we confirmed the presence of normal myelin sheath with no myelination defects or myelin reduction associated with GDAP1 defective nerves (S1D Fig).

### Lack of GDAP1 leads to loss of motor neurons and abnormal neuromuscular junctions

**Fig 3:**
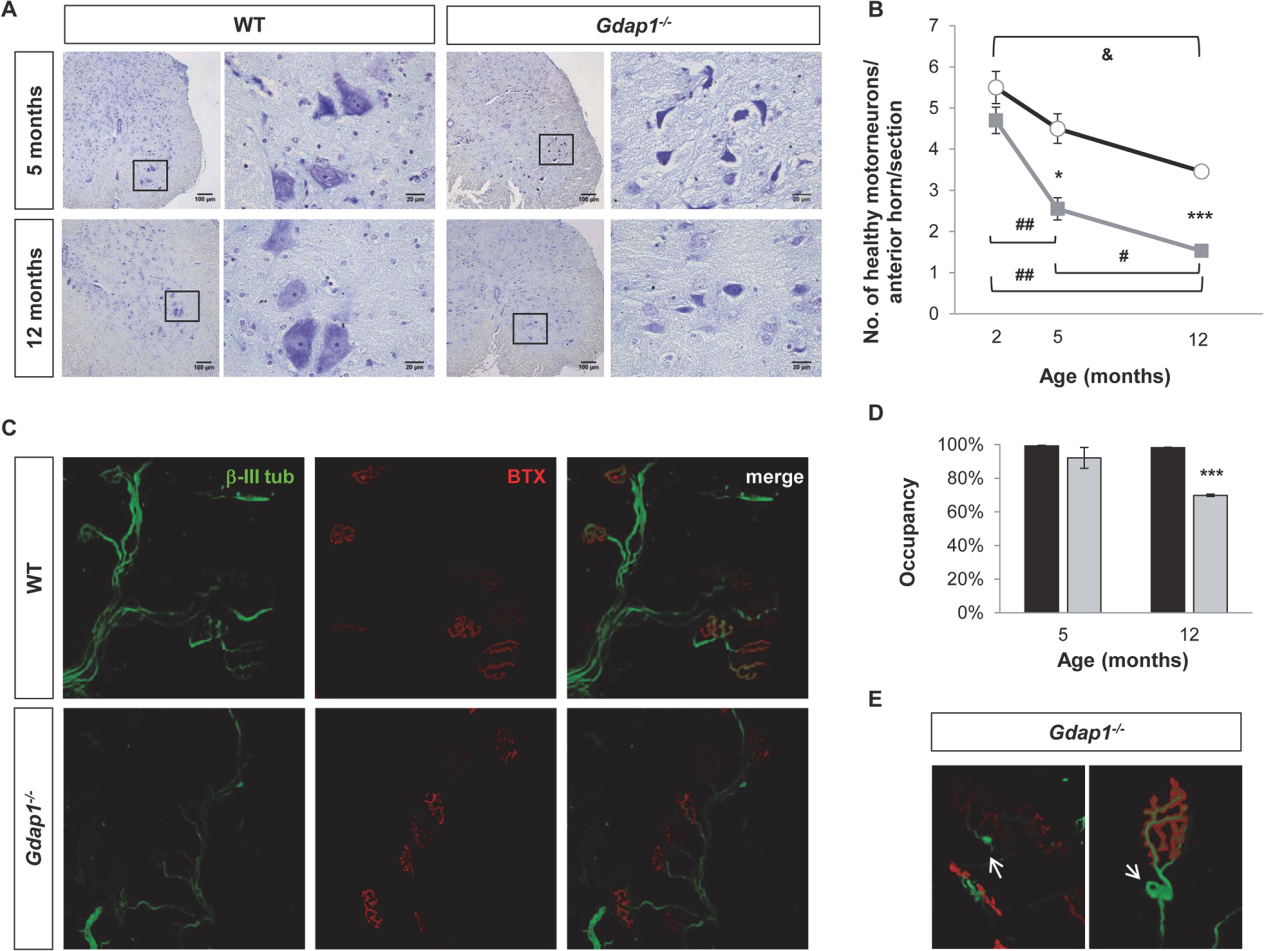
Lack of GDAP1 leads to loss of motor neurons and abnormal neuromuscular junctions. **(A)** Anterior horns from lumbar spinal cord of 5 and 12-months-old mice were stained by Nissl staining. Reduced number of MNs and evidence of chromatolysis are visible in *Gdap1* ^*-/-*^ mice. **(B)** Progressive graphics representing the number of healthy motor neurons in anterior horns per section in WT (black line) and *Gdap1* ^*-/-*^ (gray line) mice at several ages (n = 3). **(C)** Representative confocal stack images of NMJs from the gastrocnemius muscle. Axons were immunostained with anti-β-III tubulin (β-III tub, green) and the postsynaptic acetyl-choline receptor was stained with AF488-coupled α-bungarotoxin (BTX, red). **(D)** Histograms show the percentage of NMJ occupancy by terminal axons in WT (black bars) and *Gdap1* ^*-/-*^ (gray bars) mice. **(E)** Magnification of tangle-like abnormal structures (white arrows) at the terminal axons closed to the NMJ observed in *Gdap1* ^*-/-*^ mice muscles. * represents significant differences between WT and *Gdap1* ^*-/-*^ mice; **&** indicates differences between ages of WT mice; and **#** indicates differences between ages of *Gdap1* ^*-/-*^ animals (Student’s *t* test, data are presented as means ±S.E.M.).

Axonal neuropathy revealed by our electrophysiological studies in 5-months-old *Gdap1* ^-/-^ mice could be associated with defects in other motor neuron-derived structures affected by the absence of GDAP1. Thus, we examined motor neuron (MN) numbers and morphology in the anterior horn, as well as the neuromuscular junction (NMJ) structure in *Gdap1* ^-/-^ mice at different ages. MNs in the anterior horn showed cell lesions such as vacuoles and chromatolysis in *Gdap1* ^-/-^ mice at 5 and 12 months of age (Fig 3A). We found statistically significant difference in the number of healthy MNs between knockout and control mice at 5 and 12 months of age. However, while in control mice the number of healthy MNs per section underwent a progressive reduction over time (2-months vs 12-months WT animals, p-value<0.05), this process is enhanced in *Gdap1* ^-/-^ mice as indicated by the slopes of neuron loss between 2 and 5-months (*Gdap1* ^-/-^: 0.71 versus WT: 0.333). By contrast, slopes were equal between 5 and 12-months, which suggest that loss of neurons occurs early in the first months of life in the *Gdap1*-null mice (Fig 3B). These data suggest that MN degeneration in the anterior horn may contribute to the abnormalities revealed by CMAP measurements in 5-months *Gdap1* ^-/-^ mice.

Effects of pathological and functional changes in CMT diseases are related to the axonal length, so distal limb muscles are usually the first to be affected. This is the case for patients with *GDAP1* mutations, especially those carrying two recessive mutated alleles [23]. Thus, we investigated the proper occupancy and morphology of NMJs in both *Gdap1* ^-/-^ and control mice by staining axonal presynaptic buttons with an antibody against β-III tubulin and the nicotinic acetylcholine receptor with α-bungarotoxin. Occupancy of the NMJs was defined as the percentage of postsynaptic buttons that are completely filled by the nerve terminal (Fig 3C). We did not observe any differences in the NMJ occupancy at 5-months. However, whereas NMJs from 12-month-old control mice were fully occupied the percentage of occupancy in the NMJs from *Gdap1* ^-/-^ was significantly reduced (Fig 3D). In addition, we also observed abnormal tangle-like structures in terminal nerves from knockout animals that were not present in control mice (Fig 3E).

### Cellular changes in *Gdap1* ^-/-^ mice neurons

**Fig 4:**
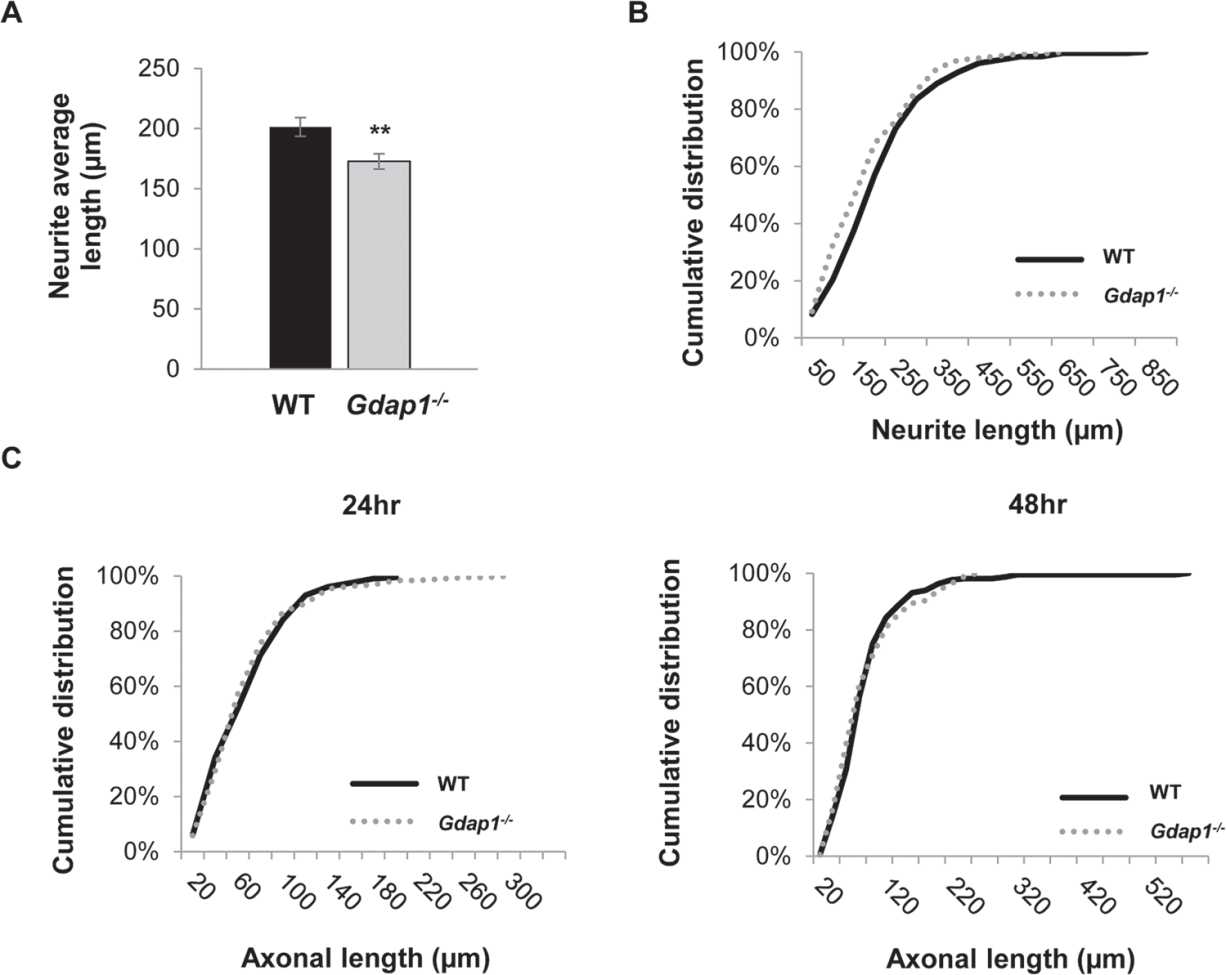
Characterization of primary sensory and motor neuron cultures from *Gdap1* ^*-/-*^ mice. **(A)** Histogram representing significant differences of the neurites average length from WT (black bar) and *Gdap1* ^*-/-*^ (gray bar) mice (graph represents means and S.E.M of 3 independent culture preparation per genotype. Student´s test **p<0.01) and **(B)** cumulative distribution of each neurite length from DRG sensory neurons cultured for 24 hours from 5-months old WT (black solid line) and *Gdap1* ^*-/-*^ (dotted gray line) mice which are significantly different (p<0.001, Kolmogorov–Smirnov test). **(C)** Cumulative distribution of axonal length from embryonic MNs cultured for 24 hours (left graph) and 48 hours (right graph) from WT (black solid line) and *Gdap1* ^*-/-*^ (dotted gray line) mice. The two plots are not significant different (p>0.05; Kolmogorov–Smirnov test. *n* = 195 axons from three independent experiments).

We decided to further investigate the consequences of the absence of GDAP1 in primary neuronal cultures. After being cultured for 24 hours, *Gdap1*-null DRG sensory neurons from 5-months old mice were able to generate neurites but we observed a significant reduction of neurite average length in sensory neurons of *Gdap1* ^*-/-*^ animals (Fig 4A) and in general longer processes in WT versus *Gdap1* ^-/-^ mice (Fig 4B). Embryonic MN cultures derived from both genotypes had similar capacity to generate axons at 24 hours (length between 200 and 250 μm). However, at 48 hours we observed substantial difference between the two genotypes (Fig 4C): whereas axons in cultures generated from the knockout mice have maximal length of around 250 μm, axons from wild-type mice showed continuous growth, some of them reaching 600 μm.

**Fig 5:**
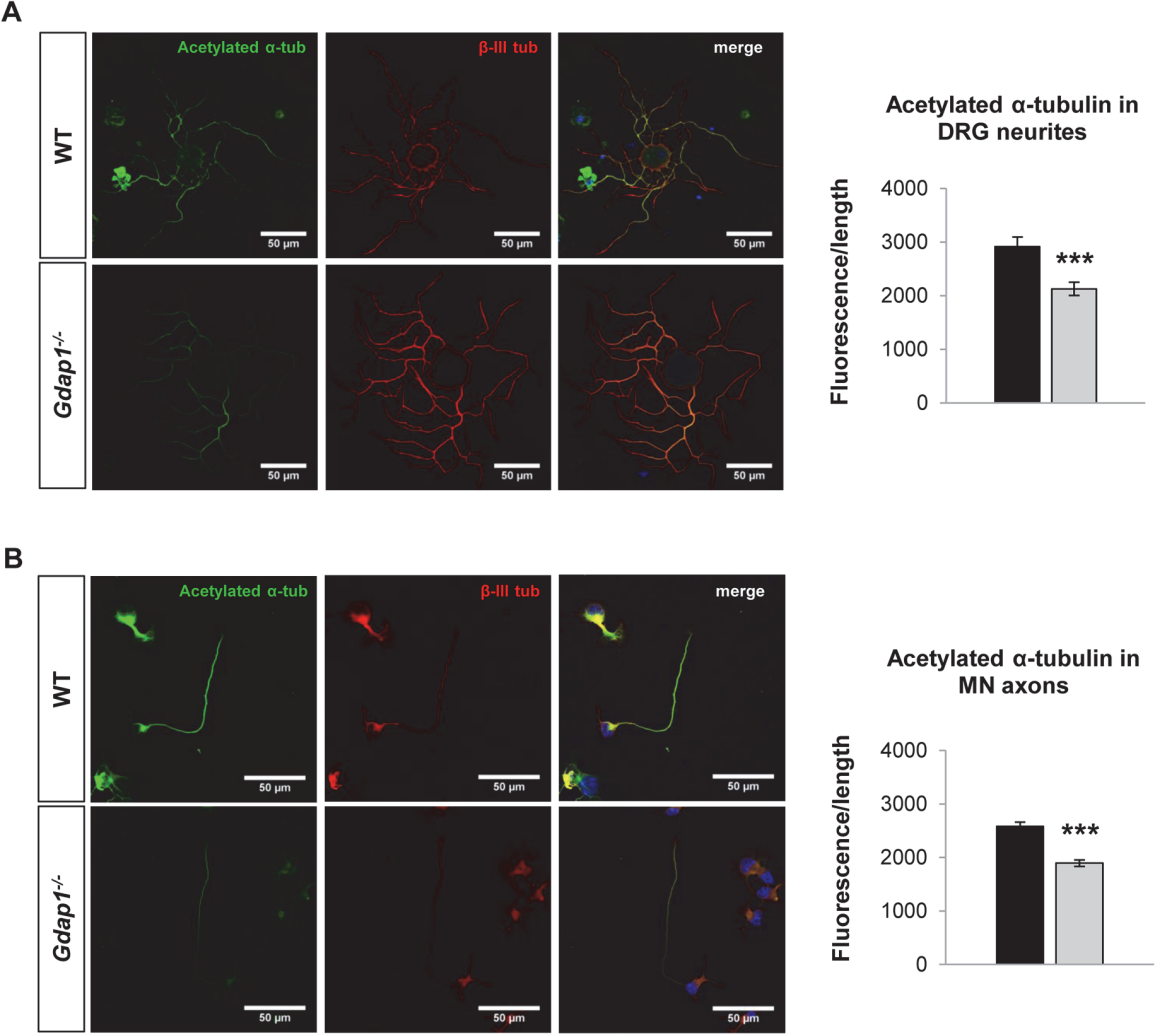
Postranscriptional modification of the tubulin cytoskeleton in primary sensory and motor neuron cultures. **(A)** DRG sensory neurons and **(B)** embryonic MNs were double-stained for acetylated α-tubulin (acetylated α-tub, green) and β-III tubulin (β-III tub, red). As indicated by respective histograms there is a significant reduction of acetylated α-tubulin in both MN and sensory neurites in *Gdap1* ^*-/-*^ mice. Graph represents means and S.E.M of 3 independent culture preparation per genotype. Student’s *t* test ***p<0.001.

We have previously demonstrated that GDAP1 interacts with vesicle transport proteins RAB6B and caytaxin, and with β-III tubulin [17,24]. Interaction with β-III tubulin prompted us to investigate the effect of GDAP1 absence on the cytoskeleton of mouse MNs and DRG neurons. Microtubules (MTs) are dynamically assembled polymers of α- and β-tubulin that are present in all eukaryotic cells. Post-translational modifications (PTMs) that occur on MT are crucial in order to maintain their dynamic stability. Many different PTMs have been reported to occur on tubulin [25]. Among them, tubulin acetylation has been widely related to neurodegenerative diseases [26], in particular with CMT [27]. Thus, we analyzed lysine-ε-acetylation of α-tubulin in a 24-hr cell culture system. We observed abnormal PTM of microtubules: α-tubulin showed significant decreased acetylation in both neurites of sensory neurons (Fig 5A) and MN axons (Fig 5B). These findings suggested a defect of the cytoskeleton associated with the absence of GDAP1 in mitochondria.

**Fig 6:**
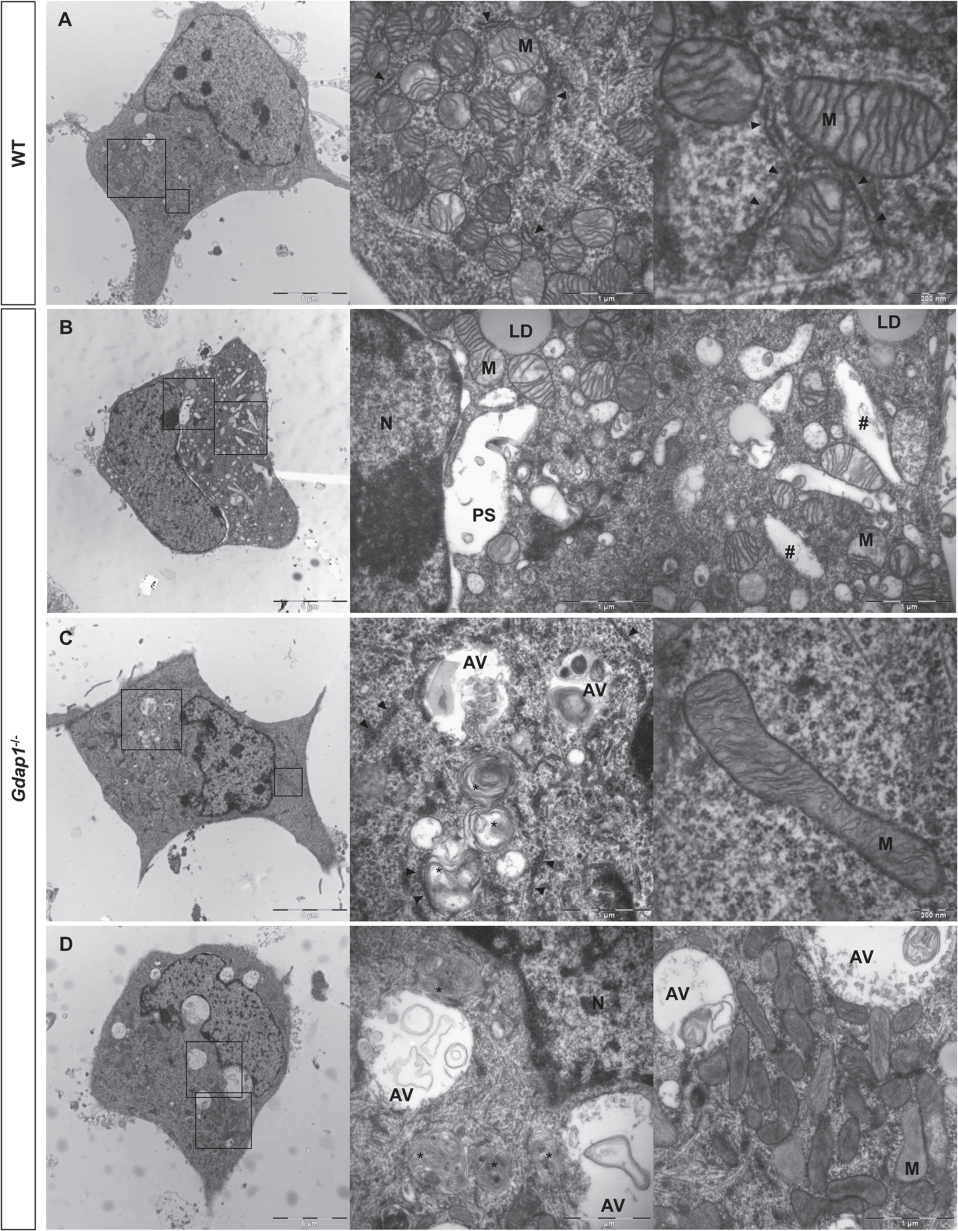
Ultrastructural analysis of embryonic motor neurons. WT **(A)** and *Gdap1* ^*-/-*^ **(B-D)** cultured embryonic MNs are shown. Medium and right panels show higher magnifications of frames of whole cell in left panels. N: nucleus; M: mitochondria; PS: perinuclear space; LD: lipid droplet; AV: autophagic vacuole; *: autophagolysosome; #: dilated endoplasmatic reticulum cisternae; arrowheads: endoplasmatic reticulum.

To get more information about the effect of loss of GDAP1 in the cell biology of neurons we investigated the cell and organelle ultrastructure in 24-hr cell cultures of both WT (Fig 6A) and knockout MNs (Fig 6B–6D). For both genotypes our cultured MNs showed neurites and putative axonal prolongations. Control MNs showed irregular and invaginated nuclei, reduced ER cisternae, large number of free ribosomes, well-defined microtubules network, and polarized mitochondrial distribution (Fig 6A). In MNs from *Gdap1* ^-/-^ mice we could distinguish abnormal organelles structures with two major patterns that may be interpreted as consecutive steps of the same pathological process: (i) neurons with dispersed round or spheroid mitochondria and apparent increase of mitochondrial metabolism, enlargement and dilatation of the nuclear envelope and perinuclear space, and significant increase number of small and medium-sized vacuoles that may correspond to dilated ER cisternae (Fig 6B); (ii) more severely affected neurons with large and tubular mitochondria with swollen cristae that are oriented along the longitudinal axis, very large vacuoles with invaginations that are originated from the nuclear envelope and from the small-medium vacuoles, phagolysosomes, and some autophagosomes; it seems that ER cisternae are more compacted with reduced internal light (Fig 6C and 6D). These observations suggest progressive cell pathology associated with the lack of GDAP1 in motor neurons. Then, we characterized in detail morphological parameters from more than 900 WT and *Gdap1*-null mitochondria (Table 1). There were no differences in the number of mitochondria per cell, surface area and perimeter of mitochondria between both genotypes. Further analysis of mitochondrial shape parameters indicated that whereas Feret diameter and roundness showed no differences, there were significant reduction of circularity and increase of aspect ratio in *Gdap1*-null mitochondria. It seems that the absence of GDAP1 induces larger mitochondria that may represent the increase of fusion activity of the mitochondrial network.

**Table 1:** Detailed morphological parameters for WT and Gdap1^-/-^ mitochondria in mouse motorneuron primary culture.

		Mean (SEM)	P value
Number of mitochondria in 100 μm			
	WT	76 (6)	0.330
	*Gdap1* ^-/-^	69 (4)	
Surface Area, μm^2^			
	WT	0.16 (0.01)	0.496
	*Gdap1* ^-/-^	0.16 (0.01)	
Perimeter, μm			
	WT	1.60 (0.04)	0.180
	*Gdap1* ^-/-^	1.66 (0.03)	
Feret's Diameter, μm			
	WT	0.58 (0.01)	0.230
	*Gdap1* ^-/-^	0.61 (0.01)	
Circularity (0–1)			
	WT	0.77 (0.01)	0.005
	*Gdap1* ^-/-^	0.73 (0.01)**	
Roundness (0–1)			
	WT	0.65 (0.01)	0.120
	*Gdap1* ^-/-^	0.61 (0.02)	
Aspect Ratio			
	WT	1.67 (0.04)	0.011
	*Gdap1* ^-/-^	1.88 (0.07)*	

Mitochondrial shape descriptors were measured in 20 WT and 30 *Gdap1* ^-/-^ motorneurons. Student’s t test was performed for normal distributed parameters (number of mitochondria, circularity, roundness and aspect ratio) and Mann-Whitney U test for those that were non-normal distributed (surface area, Feret´s diameter and perimeter). See Fig 6 for a visual representation.

*p<0.05,

**p<0.01.

### Mitochondrial defects induced by loss of GDAP1 function

**Fig 7:**
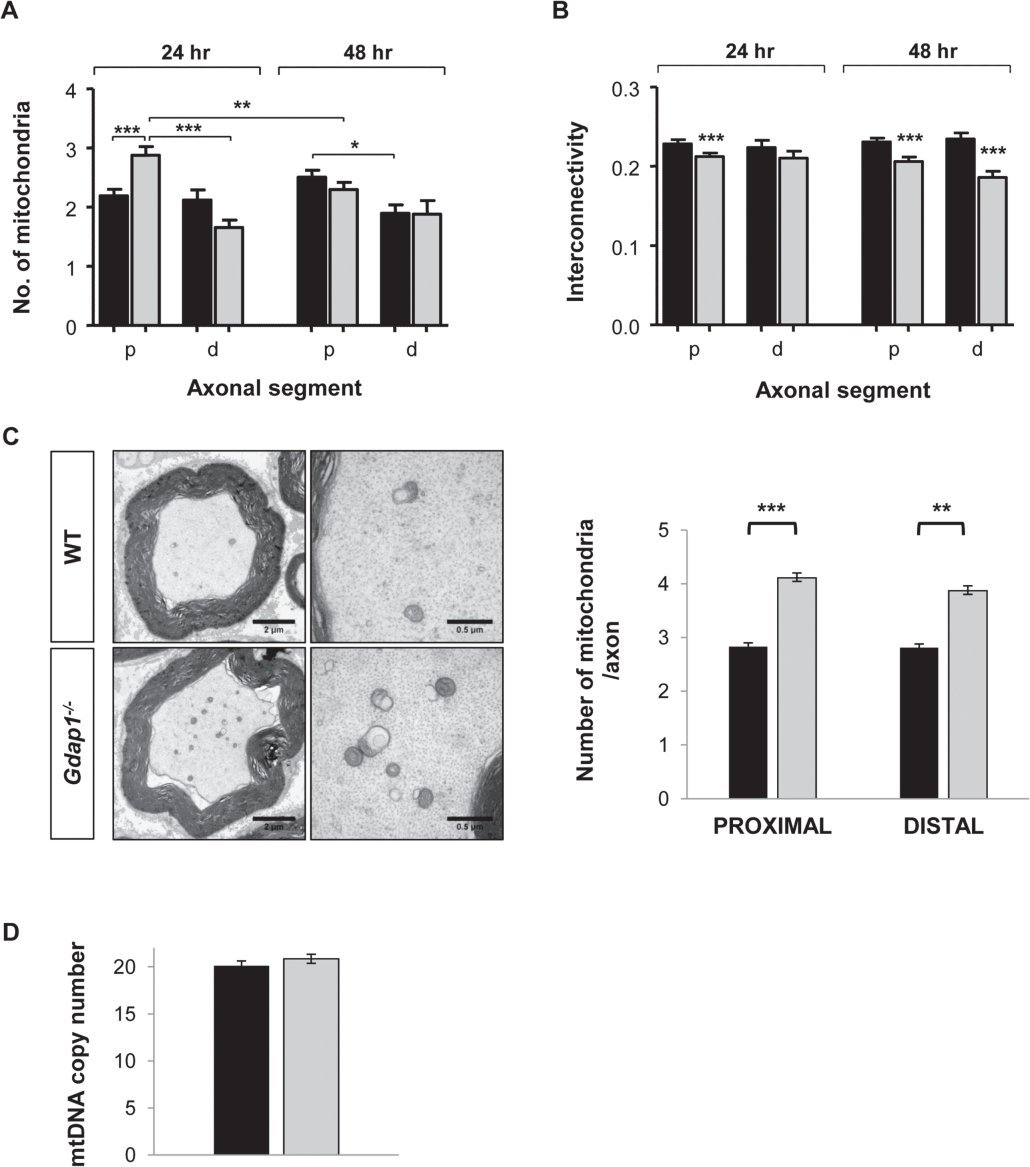
Mitochondrial quantitation and network distribution in cultured MNs and sciatic nerves from WT and *Gdap1* ^*-/-*^ mice. Number of mitochondria **(A)** and network interconnectivity **(B)** in cultured MNs are represented. The study was performed in the proximal segments (p) and distal segments (d) of WT (black bars) and *Gdap1* ^*-/-*^ (gray bars) axons after 24 hour and 48 hour of cell culture. Error bars represent S.E.M. Student’s *t* test *p<0.05, **p<0.01 and ***p<0.001 **(C)** Left panel shows semi-thin cross sections of the sciatic nerve from five months old WT and *Gdap1* ^-/-^ mice. Mitochondria are clearly visible on higher magnification images of transversal section (right panel). Mitochondrial axonal content was quantified by electron microscopy on proximal and distal cross sections of the sciatic nerve. (n = 4; Error bars represent S.E.M.; asterisks indicate significant differences between WT and *Gdap1* ^*-/-*^ mice, Mann-Whitney test, **p<0.01,***p<0.001). **(D)** Measurement of mitochondrial DNA (mtDNA) copy number in sciatic nerves.

Since *GDAP1* mutations have been associated with changes in the dynamics of the mitochondrial network and we detected morphological changes in mitochondria from somas of *Gdap1* ^-/-^ MNs (Fig 6) we decided to characterize mitochondrial phenotype further. We investigated mitochondrial parameters in both proximal (first 10 μm after neuron body) and distal (last 10 μm) segments of MN axons cultured for both 24 hours and 48 hours by detecting mitochondria as cytochrome *c* positive organelles (Fig 7A and 7B). At 24 hours WT mice showed homogenous distribution in proximal and distal axonal segments. On the contrary, in *Gdap1*-null mice the number of mitochondria was significantly increased at 24 hours in the proximal segment (Fig 7A), which could be interpreted as a delay of mitochondrial distribution along the axon in the absence of GDAP1. Mitochondrial distribution normalized after 48 hours in culture. In parallele, we observed a reduction of the mitochondrial interconnectivity that was evident in the proximal segment after 24 and 48 hours in culture and in the distal segment after 48 hours (Fig 7B). The increased number of mitochondria in the proximal segment at 24 hours and reduction of organelles interconnectivity suggests an anomalous distribution of the mitochondrial network associated with the lack of GDAP1.

We then decided to validate these data *in vivo*, by analyzing the effect of GDAP1 absence on the structure and number of mitochondria in the proximal and distal regions of sciatic nerve. Whereas mitochondrial morphology was unaffected in *Gdap1* ^-/-^ nerves, the number of mitochondrial particles was significantly increased both proximally and distally (Fig 7C). To know whether such an apparent increase could represent a true increase of the number of mitochondria or may reflect the increase of the mitochondrial mass we determined the number of mtDNA copies in sciatic nerves. We did not detect any significant difference on the mtDNA copy number, and hence mitochondrial mass, between *Gdap1* ^-/-^ and WT nerves (Fig 7D).

**Fig 8:**
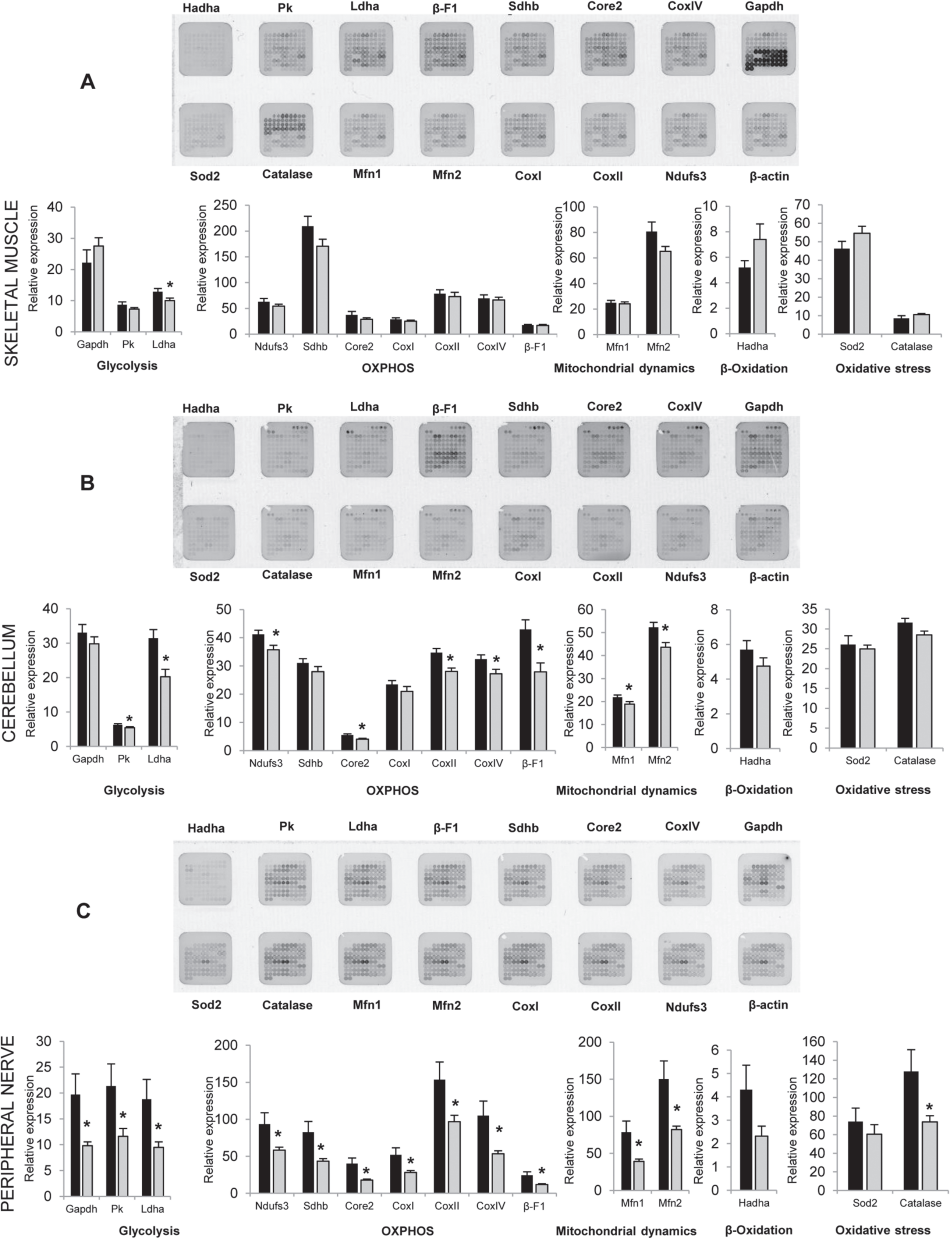
Selected metabolic protein profiling of tissues from *Gdap1* ^*-/-*^ mice. Representative RPPM for each tissue (top) and histograms (bottom) show the expression level of selected markers in various processes of the mitochondrial metabolism in skeletal muscle (**A**), cerebellum (**B**) and peripheral nerves (sciatic and axillary) (**C**) of 5-months WT (white bars) and *Gdap1* ^*-/-*^ (grey bars) mice (for printing details see S2B Fig). β-actin was used as loading control. The expression level of the different proteins was expressed relative to that found in HCT116 colon cancer cells. The means (±S.E.M) are indicated. Asterisk indicates significant differences between WT and *Gdap1* ^*-/-*^ mice (n = 10, *p<0.05, Student’s *t* test).

Motor and sensory neuropathies are frequently a consequence of mitochondrial defects associated with changes in mitochondrial respiratory chain and oxidative phosphorylation, that affect the energetic metabolism of peripheral nerves. To evaluate if similar type of changes are present in *Gdap1* ^-/-^ animals. we performed protein expression analysis on selected mitochondrial proteins at the age of 5 months. Extracts from skin, skeletal muscle, liver, brain, cerebellum, spinal cord and peripheral nerves were prepared and printed onto Reverse Phase Protein Microarrays (RPPM) to quantify the expression of relevant enzymes of glycolysis (GAPDH, PKM2, LDHA), oxidative phosphorylation [OXPHOS, Complex I (NDUFS3), Complex II (SDHB), Complex III (Core 2), Complex IV (COXI, COXII and COXIV) and Complex V (β-F1-ATPase)], mitochondrial dynamics (MFN1 and MFN2), β-oxidation of fatty acids (HADHA), oxidative stress (SOD2 and catalase) and the cytoskeleton (β-actin, used as loading control) using validated antibodies (S2 Fig). For most of the analyzed tissues (liver, skeletal muscle, brain, skin, and spinal cord) no relevant differences were observed in the expression of the proteins studied (Fig 8A,). Interestingly, cerebellum, involved in balance and motor behavior, and especially peripheral nerves (sciatic and axillary nerves) showed significant differences in protein expression (Fig 8B and 8C). Specifically, the cerebellum of *Gdap1* ^-/-^ mice revealed a diminished expression of several enzymes of glycolysis, OXPHOS and mitochondrial dynamics when compared to WT mice, suggesting a limited energy provision in this tissue (Fig 8B). Even more significant changes were observed in expression of all markers of glycolysis, OXPHOS and mitochondrial dynamics as well as catalase of oxidative stress but not HADHA of β-oxidation in peripheral nerves of *Gdap1* ^*-/-*^ mice when compared to WT mice (Fig 8C), which were confirmed by Western blot experiments in nerves (S3 Fig). This strongly suggests that a bioenergetic compromise, because of dysfunctional mitochondria, may contribute to a dysfunction of peripheral nerves and most likely cerebellum in *Gdap1* ^*-/-*^ mice. Intriguingly, differences on peripheral nerves were more significant in females, which may be related to the animal hormone environment.

### Pathogenic effects of lack of GDAP1 involve Ca^2+^ signaling

In SH-SY5Y cells we have observed that GDAP1 depletion decreases SOCE activity and impairs Ca^2+^ entry in mitochondria following activation of store-operated calcium entry [17]. This might be related to the altered distribution of the mitochondrial network within the cell associated with abnormal movement of mitochondria along the cytoskeleton towards the ER and subplasmalemmal microdomains in neuroblastoma cells having residual GDAP1 expression. With our knockout mouse model we are now able to analyze the consequences of a complete lack of GDAP1 on Ca^2+^ levels in the cytoplasm in neurons and the effect on SOCE *in vivo*.

**Fig 9:**
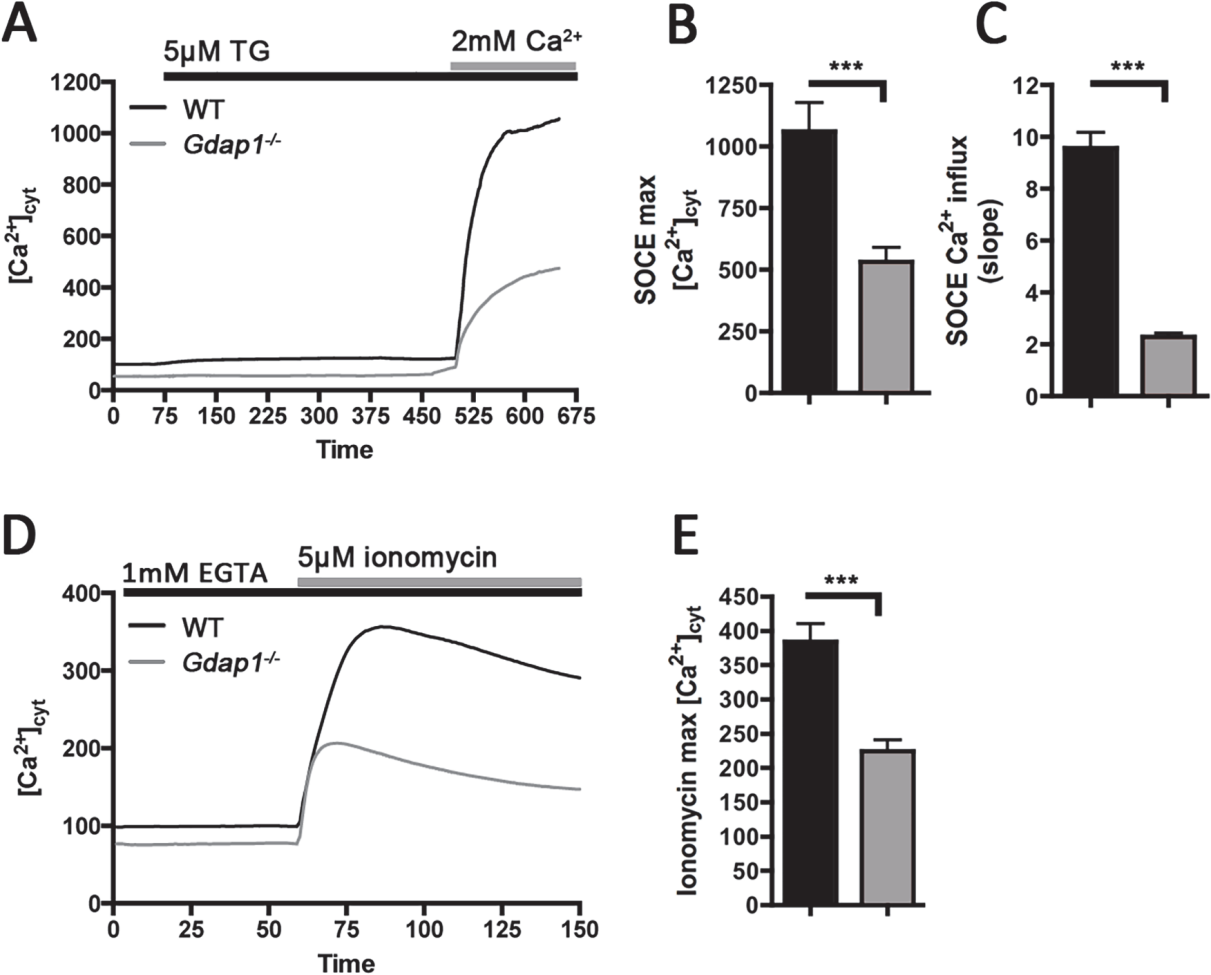
SOCE alteration in *Gdap1* ^*-/-*^ embryonic motor neurons **(A)** Fura-2 [Ca^2+^] signals of embryonic MNs from WT (black) and *Gdap1* ^*-/-*^ (grey) mice. After Ca^2+^ release from cell stores with 5 μM thapsigargin (TG) treatment during 7 min in Ca^2+^ free medium, SOCE was activated by adding 2 mM of CaCl_2_. Traces were used to obtain **(B)** maximum Ca^2+^ peak during SOCE and **(C)** SOCE Ca^2+^ influx (slope). **(D)** Fura-2 recordings of 5 μM ionomycin elicited [Ca^2+^]_cyt_ peak in Ca^2+^-free medium. **(E)** Maximum [Ca^2+^]_cyt_ peak obtained in Ca^2+^-free medium represents total amount of cytoplasmic Ca^2+^ after cell stores Ca^2+^ release. Traces were obtained averaging at least 100 cells from each genotype. Error bars represent S.E.M. (***p<0.001, Student’s *t* test).

We first studied cytoplasmic calcium ([Ca^2+^]_cyt_) transients triggered by emptying of ER-Ca^2+^ with thapsigargin (TG) in a Ca^2+^-free medium and the effect of Ca^2+^ readmission on SOCE activity in 24 hour-cultured motor neurons. Interestingly, neurons lacking GDAP1 showed significant lower [Ca^2+^]_cyt_ than WT neurons before and after ER-Ca^2+^ emptying with TG (Fig 9A). Both WT and *Gdap1* ^*-/-*^ cells responded to 2 mM Ca^2+^ by increasing [Ca^2+^]_cyt_, which suggest that SOCE mechanism is working. However, SOCE response was smaller with lower [Ca^2+^]_cyt_ peak in *Gdap1* ^*-/-*^ neurons, which may reflect the reduction of Ca^2+^ in resting conditions (Fig 9B). In addition, such reduced Ca^2+^ levels was also associated with slow entrance velocity of Ca^2+^ into the neuron (Fig 9C). Low resting levels of [Ca^2+^]_cyt_ and slow entry of Ca^2+^ after store Ca^2+^ depletion suggest that lack of GDAP1 induces a defect on the proper maintenance of intracellular Ca^2+^ concentration and homeostasis. To know if such a low cytoplasmic Ca^2+^ concentration may be due to reduced calcium stores in cells, we treated motor neurons with ionomycin, a strong ionophore that releases calcium from cellular stores. Neurons defective for GDAP1 showed significant lower increase of [Ca^2+^]_cyt_ than WT neurons, which might affect ER-Ca^2+^ release or reduction of Ca^2+^ stores (Fig 9D and 9E). These findings suggest that GDAP1 is necessary to maintain neuronal Ca^2+^ homeostasis. To know more on Ca^2+^ management within the ER, we further investigate the effect of lack of GDAP1 in the ER-Ca^2+^ physiology by measuring the calcium-handling proteins calreticulin, IP_3_R and BIP in both mouse sciatic nerve and lumbar spinal cord (S4 Fig). Whereas *Gdap1* ^*-/*-^ mice did not showed any change of the expression levels of calreticulin and BIP, the absence of GDAP1 induced upregulation of IP_3_R. This change was significant in the sciatic nerve at the age of 5 months and spinal cord at the age of 12 months. Such an upregulation might be interpreted as a specific response to the [Ca^2+^]_cyt_ depletion in MNs.

## Discussion

Mitochondria have a pivotal role in the pathogenesis of neurodegeneration [28]. Besides the classical role of defective oxidative phosphorylation in neurological disease there is increasing evidence of the relevance of mitochondrial trafficking and transport, inter-organelle communication—especially with the ER—and mitochondrial dynamics and quality control [28,29]. Peripheral nerve pathologies are observed in mitochondrial disorders and vice versa CMT has been associated with mutations in genes implicated in mitochondrial biology e.g *MFN2* and *GDAP1* [1].

Mutations in the *GDAP1* gene cause both demyelinating and axonal CMT neuropathies. Most patients are homozygous or compound heterozygous for the mutation and express a severe early-onset of autosomal recessive CMT4A [6,18] or AR-CMT2K [7,19] phenotypes respectively. Yet milder axonal dominant variant (CMT2K) has also been reported [8,9,30], which suggest different pathogenic consequences of the GDAP1 mutant protein and mechanisms of the neuropathy. GDAP1 is mainly expressed in neurons [14], but expression in Schwann cells has also been reported [11]. Thus, a major question regarding the pathophysiology of *GDAP1*-related CMT is the primary injury target in the nerve, the axon or the Schwann cell.

In order to gain insight into the cellular pathogenesis of *GDAP1* defects and disease pathophysiology we have generated and characterized a knockout mouse model of recessive forms (CMT4A/2K) of *GDAP1*-related peripheral neuropathy. We observed multiple differences in motor behavior of *Gdap1* ^*-/-*^ mice. Knockout animals showed significant reduced running time on the rotarod starting at the age of 3 months, suggesting presence of defects in the balance and motor coordination. This difference disappeared at later time-points (>9 months), likely as a consequence of the onset of age-related coordination impairments at control mice or because of the compensatory effects in CNS of the *Gdap1* gene paralogue *Gdap1l1* (S5 Fig) [31]. Gait analysis also showed changes in the motor behavior in 5- and 12-months animals as measured by the plantar footprint including stride length and angle. Similar features, even having earlier onset, have also been observed in mouse models of neurological disease involving motor neurons such amyotrophic lateral sclerosis [32,33]. Thus, the observed motor behavior deficits suggested that lack of GDAP1 leads to a peripheral neuropathy phenotype.

Reduced CMAPs amplitude in both distal and proximal nerve segments from 5-months-old mice strongly suggested the axonal nature of the neuropathy. Unexpectedly, normal number of axons and axon diameter distribution revealed no evidence of morphological axonopathy as observed in patients [19,22,23] or signs of demyelination. However, physiological damages were revealed by proteomic studies of the energetic metabolism in peripheral nerves. Limited energy supply and reduction of mitochondrial mitofusins under the *Gdap1* ^*-/-*^ background further suggested the existence of an axonal defect associated with mitochondrial abnormal function. Such functional-morphological contradictions may reflect specific response to the lack of GDAP1 in mice that could be more subtle than in human beings or reflect differences between both species related to diseased nerve length and/or natural history [23]. In contrast with the unaltered morphology of nerves, *Gdap1* ^*-/-*^ mice showed evidence of pathological changes in both motor neuron somas and the NMJs. This phenotype may be interpreted in two ways: (i) changes in motor neurons might be either a compensatory response to the abnormal function of nerves that are not altered enough to show pathological changes, or lack of GDAP1 in mice may be expressed primarily as a motor neuron disease; or (ii) both proximal lesions at neuronal somas and distal changes at NMJs may be the consequence of mitochondrial dysfunction in dendrites and distal synapsis where mitochondria are more present.

GDAP1 is located at the MOM and has originally been previously show to play a role in mitochondrial dynamics [11,12,34]. The increase in mitochondrial density observed in both proximal and distal axons of *Gdap1* ^*-/-*^ mice can be caused by various changes in mitochondrial biology: alteration in mitochondrial fission-fusion balance, increase of the total mitochondrial mass or mitochondrial transport defects. Since we observed that in sciatic nerves of *Gdap1* ^*-/-*^ mice the total mitochondrial mass is not different between WT and knockout mice, we favor the hypothesis of a defective mitochondrial dynamics. Interestingly, an increase in the number of mitochondria in peripheral axons, especially in those with diameters smaller than 3.5 μm, has also been observed in *Mfn2* transgenic mice which is a mitochondrial fusion-related protein that is also located in the MOM [35]. In addition, we also observed that the loss of GDAP1 is associated with lower interconnectivity of organelles found in primary culture of MNs, reduced axon growth capacity in both MNs and DRG sensory neurons, and a reduction of microtubule acetylation. Together, defects in the maintenance of the mitochondrial network and cytoskeleton along with abnormal energy metabolism might explain the electrophysiological changes in axons with preservation of the nerve architecture.

More recent experimental data suggest a protective role of GDAP1 against oxidative stress related to intracellular levels of glutathione [16,36]. Lopez Del Amo et al. have validated the *Drosophila GDAP1* ortholog (*Gdap1*) [36]. In this model, *Gdap1* RNAi produces progressive aggregation of the mitochondria, and eventually the presence of large elongated mitochondria in the fly thorax muscle, and at the retina mitochondria have larger size and tend to lose their peripheral localization. Moreover, we have shown that depletion of GDAP1 in the SH-SY5Y cells decreases SOCE activity and impairs SOCE-driven Ca^2+^ uptake in mitochondria, which may be linked to abnormal distribution of the mitochondria network [17]. In addition, GDAP1 is also present in mitochondrial-associated membranes (MAMs) [17] suggesting its function in the interaction between mitochondria and ER as it has also been proposed for MFN2, the gene mutated in CMT type 2A [37]. Thus, available experimental data indicate that GDAP1 may link mitochondrial dynamics, ER and Ca^2+^ homeostasis regulation. In spite of these studies, the function of GDAP1 is not well established yet. Most of the recessive mutations predict truncated proteins with loss of the C-terminal domain that anchors GDAP1 to the MOM. Missense mutations may not affect the proper protein location but rather impair mitochondrial fusion and cause mitochondrial damage [15].

Our studies on Ca^2+^ homeostasis and SOCE activation in *Gdap1* ^*-/-*^ cultured MN showed reduced [Ca^2+^]_cyt_ in both resting status and after pharmacological-mediated calcium depletion with thapsigargin or ionomycin. These findings suggest that the low levels of [Ca^2+^]_cyt_ associated with the lack of GDAP1 could represent partial depletion of cellular calcium stores and/or release defects. Reduced Ca^2+^ stores and/or abnormal ER-Ca^2+^ release may impair proper stored-operated Ca^2+^ influx as well, which in turn may affect Ca^2+^ signaling homeostasis. These data in primary mouse MN and those we previously obtained in gene-silenced human neuroblastoma cells [17,38] suggest that GDAP1 plays a relevant role on the [Ca^2+^]_cyt_ maintenance that can be, at least partially, mediated by SOCE. Thus, GDAP1 might mediate interactions between mitochondria and ER by positioning the mitochondrial network properly. The observation of large, dispersed and non-polarized mitochondria within cultured MNs in electronic microscopy experiments favors our hypothesis. Functional mitochondria are required to maintain SOCE activity [31,39]. The absence of mitochondria close to the Ca^2+^ microdomains may reduce the formation of SOCE channels between ER and plasmatic membrane and may reduce the Ca^2+^ entry operated by ER Ca^2+^ release. Calcium ions themselves control a wide number of cellular functions underlying cell signaling events. Abnormal calcium signaling has been associated with neurodegeneration [40] and increased [Ca^2+^] in the ER is observed in neurodegenerative diseases [41] but the pathological effects of reduced [Ca^2+^] in the ER is a less known phenomenon. Chronic depletion of cellular Ca^2+^, either in ER or cytosol or both, affects the fluctuations and signaling of calcium ions and may induce pathological changes in mitochondria and ER and their physiological interaction which in turn may lead to the unfolding protein response (UPR) and activate the ER-stress-induced apoptosis pathway [42]. Difficulties to maintain the Ca^2+^ concentrations in cellular compartments and the abnormal cellular response may explain the progressive phenotype and neuropathology observed in our *Gdap1* ^*-/-*^ mouse model.

Recently, Niemann *et al*. [43] reported an age-related hypomyelinating peripheral neuropathy in a *Gdap1* knockout mouse model generated by deletion of exon 5. These knockout mice developed a late-onset neuropathy with reduced nerve conduction velocity and changes in the nerve myelination. Morphometric studies revealed hypomyelination in 19-months-old knockout mice with no detectable axonal loss. In addition, sciatic nerve injury of knockout animals at the age of 2 months led to a less effective remyelination as compared with WT animals. The authors also found reduction in the CMAP amplitudes that may suggest an axonal component in the neuropathology phenotype. In contrast, our *Gdap1* ^*-/-*^ mouse model expressed an earlier onset in neuropathy-related signs as indicated by motor behavior abnormalities at the age of 3 months and electrophysiological changes in 5-months-old animals (S1 Table). Since the same technological approach was used to generate knockout animals, the major genetic difference is the ablation of exon 1 in our case versus exon 5. In both cases GDAP1 protein is absent on western blot analyses. However, we do not have sufficient information about consequences on transcription and RNA biology. These authors propose that mutations in *Gdap1* lead to mild, persistent oxidative stress in the peripheral nervous system, which may be compensated in the central nervous system (CNS) by translocation of GDAP1L1 from the cytosol to mitochondria. We have studied *Gdap1l1* expression in our *Gdap1*-null mouse, but we did not observe differences in brain, spinal cord and DRG between WT and *Gdap1*-null mice. (S5 Fig, S1 Text). However, the strong expression of *Gdap1l1* in brain of *Gdap1*-null mice, as in WT animals, may protect the brain structures from any pathological effect caused by the lack of GDAP1.

In conclusion, our data indicate that the lack of GDAP1 in mice lead to abnormalities of calcium homeostasis and changes in the axonal and neuronal physiology of the mitochondrial network and the ER. These results provide new insight into neuropathy caused by the loss of function *GDAP1* mutations. The effects of *GDAP1* recessive missense and dominant mutations on calcium signaling and the interaction with mitochondria and ER require further studies.

## Materials and Methods

### Generation of the *Gdap1* knockout mouse model

Vector construction and targeted knockout strategy was designed together with genOway (Lyon, France), where mice were generated. The genomic region of murine *Gdap1* locus was isolated from a 129Sv library/Pas, called rTgV, developed at genOway. The BAC clone collection rTgV was screened by PCR. The first pair of primers (sense: 5`-CAG GGG AAC ATA ATC TGT GAG GAG GC-3 '; antisense: 5'-TCA CTA CTG GTG GTT CTT GTC AGC GC-3') amplifies a genomic fragment 409 bp gene GDAP1 upstream of exon 1. The second pair of primers (sense: 5'-GCG ACG GAA AAG CTC TAC CCT TAC C-3'; antisense: 5'-ACT GCA GTA GCA CTT GAG TGG CAG G-3') amplified a pb 553 of exon 2 and intron 2 *Gdap1* genomic fragment. This molecular design led to the identification of a single BAC clone (# 2956A2), renamed clone FPA1-rTgV. DNA sequencing showed the Gdap1 homology regions of interest in that specific clone, which was used subsequently to isolate both FPA1-Long arm (5.7 kb) as FPA1-Short arm (1.8 kb) of target vectors. The target vector, FPA1-HR, contained two inserted *lox*P sites flanking exon 1, the neomycin positive selection gene flanked by FRT sites and the presence of diphtheria toxin A (DTA) as a negative selection marker. Robust PCR screening strategy and Southern blot for detection of homologous recombination were designed. The FPA1-HR 129SvPas construct was transfected into ES cells according to standard electroporation procedures. Positive selection was started 48 h after electroporation, by adding 200 mg/ml G418 (Life Technologies, Inc.). 179 resistant clones were isolated and amplified in 96-well plates. ES cell clones were screened by PCR to verify homologous recombination at the 3 'end of *Gdap1* locus: sense 5'-GCC ACT CTC CAG ATG TTG AAA GGA G-3'; antisense 5'-TCA CTA CTG GTG GTT CTT GTC AGC GC-3'. a product of approximately 2.1 kb was expected. Of 179 clones tested, 3' homologous recombination was observed in 8 clones, which were verified by Southern blot analysis of 3' and 5' recombination events in *Gdap1* locus. Two recombinant clones were microinjected into C57BL/6 blastocysts, giving rise to five male chimeras with significant contribution of ES cells (positive agouti color) were characterized. These male mice were bred with C57BL/6 Flp deleter female mice in order to cause cleavage of the germline neomycin selection cassette. Genotyping by PCR and Southern blot of pups derived from F1 breeding allowed identification of 6 heterozigous *Gdap1* floxed mice heterozygous (*Gdap1* ^*+/flox*^). To generate a germline deletion of exon 1 female mice *Gdap1* ^*+/flox*^ mated with male C57BL/6 expressing CMV-Cre recombinase. Such a breeding allowed us the generation of heterozygous constitutive *Gdap1* knock-out mice (*Gdap1* ^*+/*-^ mice). Heterozygotes were matched to obtain homozygous *Gdap1* ^*-l-*^ and *Gdap1* ^*+/+*^ littermate mice that were subsequently amplified by intercrossing for experimental work. All mice were maintained at 21 ± 2° C in cycles of 12 h light/dark with food and water *ad libitum*.

#### Genotyping

Genotyping was performed on tail tip DNA extracted with Wizard SV Genomic DNA purification System (Promega) according to the manufacturer’s instructions. PCR was performed using Expand High Fidelity PCR System (Roche Diagnostics) using the following primers: Forward: 5’-CCT TGT TTC TCA TCT ACT CCT ATT ATC CGT AGG-3’ and Reverse: 5’-GGA ACC CCT TCT CTC ACT TTC CAG G-3’

#### Western blotting

Snap frozen on dry ice tissues were homogenized in lysis buffer (50mM Tris HCl pH 7.4, 1.5 mM MgCl_2_, 5 mM EDTA, 1% Triton X-100, 50 mM NaF, 1mM NA_2_VO_3_) containing protease inhibitors (Roche). Tissue lysates (30μg) were collected and resolved in sodium docecyl sulfate polyacrylamide gels and transferred onto polyvinylidene difluoride Immobilon-P transfer membrane filters (Millipore, Billerica, MA, USA) using an Amersham Biosciences (Piscataway, NJ, USA) semidry Trans-Blot according to the manufacturer's instructions. The membranes were blotted with rabbit anti-GDAP1 and rabbit anti-β-actin (Sigma-Aldrich), rabbit anti-calreticulin, rabbit anti-β-tubulin, rabbit anti-BiP and rabbit-IP3 Receptor (Cell Signaling) antibodies. Blots were developed using the ECL Prime chemiluminiscent substrate (GE Healthcare Amersham).

### Motor coordination behavior and electrophysiology

#### Rotarod test

Motor performance and balance were tested using an accelerating rotarod (UGO Basile Accelerating Rotarod). Each mouse underwent for 4 days the same procedure. The first 2 days were used to train the mice (five sessions of 1 min each, walking at 4 r.p.m.). The test sessions were run on day 3 and 4. Each test day, 2 series of 3 trials with a 2 hours rest period between the 2 series and a 15 min rest period between consecutive trials was performed. During the test the speed of the rotarod was accelerated from 4 to 40 r.p.m. over a 5 min period. A different group of animals was used for each time point of the study.

#### Gait and footprint analyses

Mice crossed an illuminated alley, 70 cm length, 9 cm width, and 6 cm height, before entering a dark box at the end. Their hindpaws were coated with nontoxic water-soluble ink and the alley floor was covered with sheets of white paper. To obtain clearly visible footprints, at least 3 trials were conducted. The footprints were then scanned and analyzed with ImageJ software (NIH). Four variables were considered: print length (PL), which is the distance from the heel to the third toe, toe spread (TS), which is the distance from the first to the fifth toe, stride length (SL) as the distance between initial contacts of the same paw in one complete stride and stride angle (SA) which is the angle between the lines connecting the first toe of footpads from each side of the stride.

#### Motor nerve conduction velocity and compound muscle action potential

Animals were anesthetized with a mixture of 10 mL/g Ketanarkon 100 (1 mg/mL; Streuli) and 0.1% Rompun (Bayer) in phosphate buffered saline (PBS) and fixed in the prone position. Motor nerve conduction velocity (MNCV) and compound muscle actions potential recordings in sciatic nerve were performed as described previously [44].

### Histological features

#### Motor neuron counting

Mice were sacrificed by gentle cervical dislocation. The lumbar region of the spinal cord (L1–L5) was processed for paraffin embedding. 300 serial cross sections (10 μm thickness) of the lumbar spinal cords were made (3000 μm or 3 mm total length), among which every fifth section (50 sections examined per animal) was processed and Nissl-stained, as reported previously [45]. All cells were counted within the ventral horn below an arbitrary horizontal line drawn from the central canal. The sections were analyzed at a 40x magnification in the anterior horn (either left or right) for the presence of all neurons in that region. Morphological criteria were also employed, so that only large polygonal neurons in which the nucleolus was clearly visible at high magnification were included in the counts.

#### Neuromuscular junctions analysis

Preparation of the gastrocnemius muscles for immunohistochemistry was performed as described [46] with some modifications. Muscles were stained with α-Bungarotoxin-tetrametylrhodamine (10 μg/ml, Sigma) in PBS for 20 min for acetylcholine receptor (AChR) clusters. Rabbit anti-β-III tubulin (1:500, Sigma-Aldrich) in blocking solution was applied overnight at RT. As secondary Alexa 488-conjugated polyclonal anti-rabbit (1:100, Invitrogen) was diluted in the blocking solution and applied for 5 h at RT. Neuromuscular endplates of *Gdap1* ^*+/+*^ and *Gdap* ^*-/-*^ mice were analyzed with a Leica SP8 confocal microscope for morphological analysis.

#### Sciatic nerves morphometric analysis

Sciatic nerves from *Gdap1* ^*+/+*^ and *Gdap* ^*-/-*^ 5 months-old mice were dissected and processed as reported elsewhere [44].

### Profiling of metabolic proteins in *Gdap1* ^*-/-*^ mouse tissues

#### Preparation of tissue extracts

Five females and five males 5-months old *Gdap1* ^−/−^ mice and the same number of age-matched wild type animals were sacrificed and tissues were collected (brain, cerebellum, spinal cord, liver, skin, skeletal muscle, and sciatic and axillary nerves). ~ 0.2 g of frozen tissue powder was extracted with 300 μl of 50 mM Tris/HCl, pH 8, containing 100 mM NaCl, 1 mM DTT, 1% (v/v) Triton X100, 0.1% SDS, 0.4 mM EDTA and a cocktail of protease (Roche) and phosphatase (Sigma-Aldrich) inhibitors. Tissue samples from the mix of the two peripheral nerves (~10 mg) were extracted in 30 μl of the extraction buffer. After protein extraction, samples were centrifuged (15,000 g) at 4°C for 30 min. The protein concentration in the supernatants was determined with the Bradford reagent (Bio-Rad Protein Assay) using BSA as standard. Aliquots of the supernatants were stored at -80°C until used.

#### Printing and processing of Reverse Phase Protein Microarrays (RPPM)

Samples from mice biopsies were processed and diluted in PBS to a final protein concentration of 0.75 μg/μl before printing. The RPPM analysis was performed as described elsewhere [47] and in S2 Fig.

### Real-time polymerase chain reaction assay for mitochondrial DNA copy number

Mitochondrial DNA copy number quantification was based on the mouse tissue-adapted ND1/ND4-quantification method [48]. Briefly, we quantified the mitochondrial 16S gene, starting at position mt2469. The following primer/probe combinations were used for real-time PCR with LightCycler 480 Probes Master Mix (Roche). Custom primer sequence for 16S: forward primer: 5' AAT GGT TCG TTT GTT CAA CGA TT 3', reverse primer: 5' AGA AAC CGA CCT GGA TTG CTC 3', and probe: FAM-5' AAG TCC TAC GTG ATC TGA GTT 3'-MGB (Roche). Genomic DNA of a nuclear housekeeping gene, ANG1 (Assay AppliedBiosystems: Mm00833184_s1) was also quantified using the same amount of DNA input. Relative mitochondrial DNA copy number was determined by comparing 16S gene to the nuclear endogenous control gene, also normalized to genomic DNA from blood.

### Dorsal root ganglia (DRG) neuron cultures

Wild-type and *Gdap1* ^*-/-*^ 5-month-old mice were sacrificed and their lumbar DRGs dissected out and collected in L15 media. Ganglia were incubated with 0.2% collagenase (30 min at 4°C and 1 h at 37°C; GIBCO) followed by 0.05% trypsin (30 min at 37°C; GIBCO) and 1% DNase (5 min at RT; Sigma) treatments, and further dissociated by passing several times through a Pasteur pipette. After washing in F-12 medium, cells were diluted in F-12 medium supplemented with 200 mM glutamine, 60 ng/ml progesterone, 16 μg/ml putrescine, 400 ng/ml L-thyroxine, 38 ng/ml sodium selenite, and 340 ng/ml triiodothyronine, 35% Albumax II, 10 μg/ml penicillin, 10 μg/ml streptomycin and 25 μg/ml amphotericin B. Neurons were plated at a density of 2000 cells/cm^2^ on 0.05% poly-DL-ornitine (overnight at room temperature; Sigma-Aldrich) and 20 μg/ml laminin (4 h at 37°C; Sigma-Aldrich) precoated 13 mm glass coverslips and maintained in culture at 37°C in a humidified incubator under 5% CO_2_. For immunolabelings, DRG cultures on coverslips were fixed in 4% paraformaldehyde (PFA) in PBS for 20 min, followed by 3 rinses in PBS, and an incubation with 3% horse serum and 0.2% Triton X-100 in PBS (blocking buffer) for 30 min at room temperature. Coverslips were then incubated for overnight at 4°C with monoclonal rabbit anti-β-III tubulin (1:1000; Sigma-Aldrich). After several rinses in PBS, coverslips were incubated for 1 h at room temperature, with fluorochrome-labeled secondary antibody alexa-488 (1:400; Sigma-Aldrich). Finally, coverslips were rinsed with PBS again and mounted in DAPI-fluoromount G. For quantification of various parameters of neurite outgrowth, fluorescent images from a Leica microscope were taken, and morphological measurements were performed using the plugin Neuron J from the ImageJ software. A neurite was defined as a process that measures at least the length of one cell body and stains positively for neurofilament protein. The length of the longest neurite from the cell body to its distal end were traced and measured for each neuron in order to calculate the average length (300 cells were measured at each of 3 independent experiments).

### Embryonic motor neuron (MN) cultures

MN cultures were prepared from 13.5 embryonic day (E13.5) mouse spinal cord as described previously [49–51], but with minor modifications. Briefly, mouse embryo spinal cords were dissected and the dorsal half removed. Ventral spinal cords were dissociated mechanically after trypsin treatment (0.025% trypsin in HBSS), and collected afterwards under a 4% bovine serum albumin cushion. The largest cells were isolated by centrifugation (10 min at 520 g) using iodixanol density gradient purification. The collected cells were finally suspended in a tube containing MN complete medium: Neurobasal (Life technologies) supplemented with B27 (Life technologies), 2% horse serum, 1x glutamax (Life technologies), and a cocktail of recombinant neurotrofins: 1 ng/mL BDNF; 10 ng/mL GDNF, 10 ng/mL CNTF, and 10 ng/mL HGF (PreProtech). 10 μM AraC (Sigma-Aldrich) was added to the culture medium to limit the growth of non-neuronal cells. Isolated MNs were plated on poly-D-lysine/laminin-coated surfaces as described previously [52], and grown in a 5% CO_2_ incubator at 37°C. For long-term experiments, media was changed every 2–3 days. Cultured MNs were clearly identified by immunofluorescence using SMI-32 (Covance) and HB9 (Millipore) antibodies or by morphological criteria.

#### Mitochondrial morphology and distribution in MN cultures

Isolated MNs (2000 cells) plated on glass coverslips were fixed 24 and 48 h after plating with freshly prepared 4% PFA for 20 min at 37°C. Cultures were first blocked with PBS containing 4% BSA, 0.5% Triton X-100 for 1h at RT. Subsequently, cultures were incubated for 1h with the following primary antibodies: mouse anti-SMI-32, a non-phosphorylated neurofilament-H (Steinberger Monoclonals), rabbit anti-β-III tubulin (Sigma-Aldrich), sheep anti-Hb9 (Abcam) and mouse anti-cytochrome c (Abcam). Cells were then incubated with the secondary antibodies Alexa-488 and Alexa-633 for 1h at RT and counterstained with dapi for nuclei visualization. Images were captured on the detector assembly of a DMI 6000 microscope equipped with a Leica-TSC-SP8 laser scanning confocal imaging system and with a 63x 1.4 oil immersion objective. For confocal *z*-axis stacks, series of stack images (1024x1024) separated by 0.5 μm along the *z*-axis were acquired to visualize mitochondria. 3D reconstructions of the stacks were performed with the VolumeJ plugin of ImageJ (N.I.H., USA). Mitochondrial morphology analysis was performed as previously described [53]. We evaluate the number of mitochondria and the interconnectivity index (area/perimeter ratio). To evaluate the mitochondrial distribution along the axons, images were taken at 0,75x magnification and the projections were analyzed in two separated segments (proximal, 10 μm closer to the soma and distal, 10 μm from to the axonal cone). Axonal length was determined with the NeuronJ plugin of ImageJ. Al least 50 cells were analyzed in three independent experiments.

For Transmission Electron Microscopy (TEM) isolated embryonic MNs were seeded onto poly-D-lysine/laminin-coated Lab-Tek chamber slides of 2 wells (Nalge Nunc International), fixed after 24 h of culture in 2.5% glutaraldehyde for 1 h at 37°C and processed for TEM as previously reported by Voliani et al. [54]. Two non-overlapping regions (16.500 magnification) containing numerous mitochondria were selected for each motorneuron. Mitochondrial shape descriptors and size measurements were obtained using Image J (version 1.48a, National Institutes of Health, Bethesda, MD) by manually tracing only clearly discernible mitochondria on TEM micrographs [55]. Surface area (mitochondrial size) is reported in squared micrometers; perimeter in micrometers; Feret’s diameter represents the longest distance (μm) between any two points within a given mitochondrion; circularity [4 (surface area/perimeter2)] and roundness [4 (surface area)/ (major axis2)] are two-dimensional indexes of sphericity with values of 1 indicating perfect spheroids; and aspect ratio (AR) is computed as [(major axis)/(minor axis)] and reflects the “length-to-width ratio”. Computed values were exported to Microsoft Excel. Analysis was performed at least on 900 mitochondria per genotype from independent culture preparations (n = 2 WT and n = 4 *Gdap1* ^-/-^).

### Quantitative microtubule composition in neuron cultures

For quantitative analyses, isolated neurons plated on glass coverslips were fixed 24 and 48 h after plating with freshly prepared PHEM (60 mM PIPES, 25 mM HEPES, 5 mM EGTA, 1 mM MgCl_2_, pH 7.4), 4% PFA for 20 min at 37°C, washed with PBS and then activated with NH_4_Cl for 5 min. Cells were then blocked for 45 min at RT prior to the double-staining for mouse anti-acetylated α-tubulin (Ac-tub) and rabbit anti-β-III tubulin (β-III tub)(Sigma-Aldrich). The blocking solution consisted of 10% fetal bovine serum (FBS) and 0.1% Triton X-100 in PBS. As secondary antibodies we used an anti-mouse-Alexa488 and an anti-rabbit-Alexa633. Cells were finally counterstained with dapi for nuclei visualization and kept at 4°C for 48h prior to image capture. For confocal *z*-axis stacks, stacks of 10 images separated by 0.2 μm along the *z*-axis were acquired using the appropriate color channel on the detector assembly of a DMI 6000 microscope equipped with a Leica-TSC-SP8 laser scanning confocal imaging system and with an 63x 1.4 oil immersion objective. Z-stacks at each point were collapsed to maximum projections and 3D reconstruction and volume rendering of the stacks were performed with the appropriate plugins of ImageJ (N.I.H., USA). The acetylated α-tubulin was measured with ImageJ software using stacks (6 slides, 0.21 μm each one) with the maximum projection. Results were expressed as the acetylated α-tubulin fluorescence intensity through the neurite or axon.

### Measurement of [Ca^2+^]_cyt_

Cytosolic Ca^2+^ imaging with Fura-2 was performed as described by [56]. MNs were isolated from 13.5 days embryos from each genotype as described previously. Cells were plated onto 24 mm round coverslips and, after 24 h, loaded with Fura-2AM by incubation in 30 mM D-glucose Ca^2+^-free HCSS with 5 μM Fura-2AM and 50 μM pluronic F.127 acid (Sigma-Aldrich), for 30 min at 37°C, and rinsed with HCSS, 2 mM CaCl_2_, for 30 min. Regions of interest (ROIs) were selected by morphology taking into account only motorneurons. Fluorescence (emission 510 nm) ratio of Ca^2+^-free (F380) to Ca^2+^-bound probe (F340) was analyzed using Metafluor for Leica developed by Metamorph (Universal Imaging). Analysis of SOCE was done using a standard protocol, depleting ER-Ca^2+^ using 5 μM of TG and inducing SOCE with 2 mM of CaCl_2_. The analysis of total Ca^2+^ content was carried out using 5 μM of Ionomycin (Sigma-Aldrich) in Ca^2+^-free HCSS plus 1 mM EGTA. Each experiment was plotted and calibrated in terms of [Ca^2+^].

### Statistical analyses

Calculations and statistical analysis were performed using Excel (Microsoft Corporation, Redmond, WA), Statgraphic statistical software and Prism software (GraphPad Software, Inc., San Diego, CA). The specific test applied in each case is indicated in the figure legend or the text. Differences were considered statistically significant when *p*<0.05, with a confidence limit of 95%.

### Ethics statement

The experimental research performed in animals has been approved by the Bioethics Committee of the Consejo Superior de Investigaciones Científicas (CSIC) and the Animal Experimentation Ethics Committee of the Centro de Investigación Príncipe Felipe (CIPF).

## Supporting Information

S1 Fig Characterization of sciatic nerve morphology. **(A)** Semi-thin cross sections of sciatic nerves from 5-months WT and *Gdap1* ^*-/-*^mice. No differences between genotypes were found analysing numbers of fibers in 2 and 5 months old mice **(B)**. Similarly, at proximal and distal sciatic nerves axon size distribution **(C)** and g-ratio **(D)** show no differences between 5 months old control and *Gdap1* ^*-/-*^ mice.
(TIF)

S2 Fig Validation of the antibodies used in the study (A) and printing scheme of RPPM (B). **(A)** 20 μg of tissue extracts (brain, B; heart, H) were fractionated on 4M urea SDS-PAGE gels, blotted against the indicated antibodies and processed for western blotting. Only antibodies that recognize a single protein band of the expected molecular mass were used in the study. The migration of molecular mass markers is indicated to the left. (**B)** Shows the details of the scheme of printing of RPPM. One μl samples were spotted in duplicate. Yellow boxed: negative controls of BSA; Green boxed: IgGs spotted as positive control of the secondary antibody used; Blue boxed: standard curves of HCT116 cells; Red boxed: tissue samples. The upper and lower red boxed samples correspond to different tissues. The blue plot illustrates the linear correlation that exists between the fluorescence intensity (arbitrary units, a.u.) and the amount of protein in HCT116 cell lysates. Note the absence of signal in BSA samples (yellow) and the linear increase response of the secondary antibody towards increasing content of spotted IgGs (green). Protein concentrations in the biopsies were calculated according to the fluorescence intensity obtained
(TIF)

S3 Fig Western blots show the expression level of some of the proteins studied by RPPM. 25 μg of tissue extract from peripheral nerves (sciatic and axillar) of two wild type (WT1, WT2) and two Gdap1-KO (KO1, KO2) 5-months mice were fractionated on SDS-PAGE gels, blotted against the indicated antibodies and processed for western blotting. Note the significant reduction in the expression of LDHA, COXII and MFN2, and the presence of changes in the expression of the mitochondrial SOD2, consistent with the findings reported in RPPM (Fig 8). Representative blot of citoeskeletal tubulin as loading control.
(TIF)

S4 Fig Effect of GDAP1 absence at *Gdap1* ^*-/-*^ mice tissues. Western blot analyses of Calreticulin, BIP and IP3R in sciatic nerve **(A)** and lumbar spinal cord **(B)** biopsies of WT and *Gdap1* ^*-/-*^mice. Histograms show normalized intensities measured by densitometry and calculated relative to β-actin. A representative blot is shown below each graph. Error bars are SEM. *p* values were calculated using Student's *t* test (n = 6. *p<0.05).
(TIF)

S5 Fig Ablation of *Gdap1* does not change *Gdap1l1* expression. Expression pattern of *Gdap1* paralogue gene *Gdap1l1* by semi-quantitative reverse transcriptase PCR. No differences were observed between WT and *Gdap1* ^*-/-*^ mice tissues.
(TIF)

S1 Table Genetic and phenotype comparative analysis between *Gdap1* knockout mice from our study and Niemann et al. (DOCX)

S1 Text Supplementary Materials and Methods. (DOCX)
