# Supplementary figures and images for: Lack of GDAP1 Induces Neuronal Calcium and Mitochondrial Defects in a Knockout Mouse Model of Charcot-Marie-Tooth Neuropathy
Source: PLoS Genet. 2015 Apr 10;11(4):e1005115. doi: 10.1371/journal.pgen.1005115 (PMC4393229; doi:10.1371/journal.pgen.1005115)

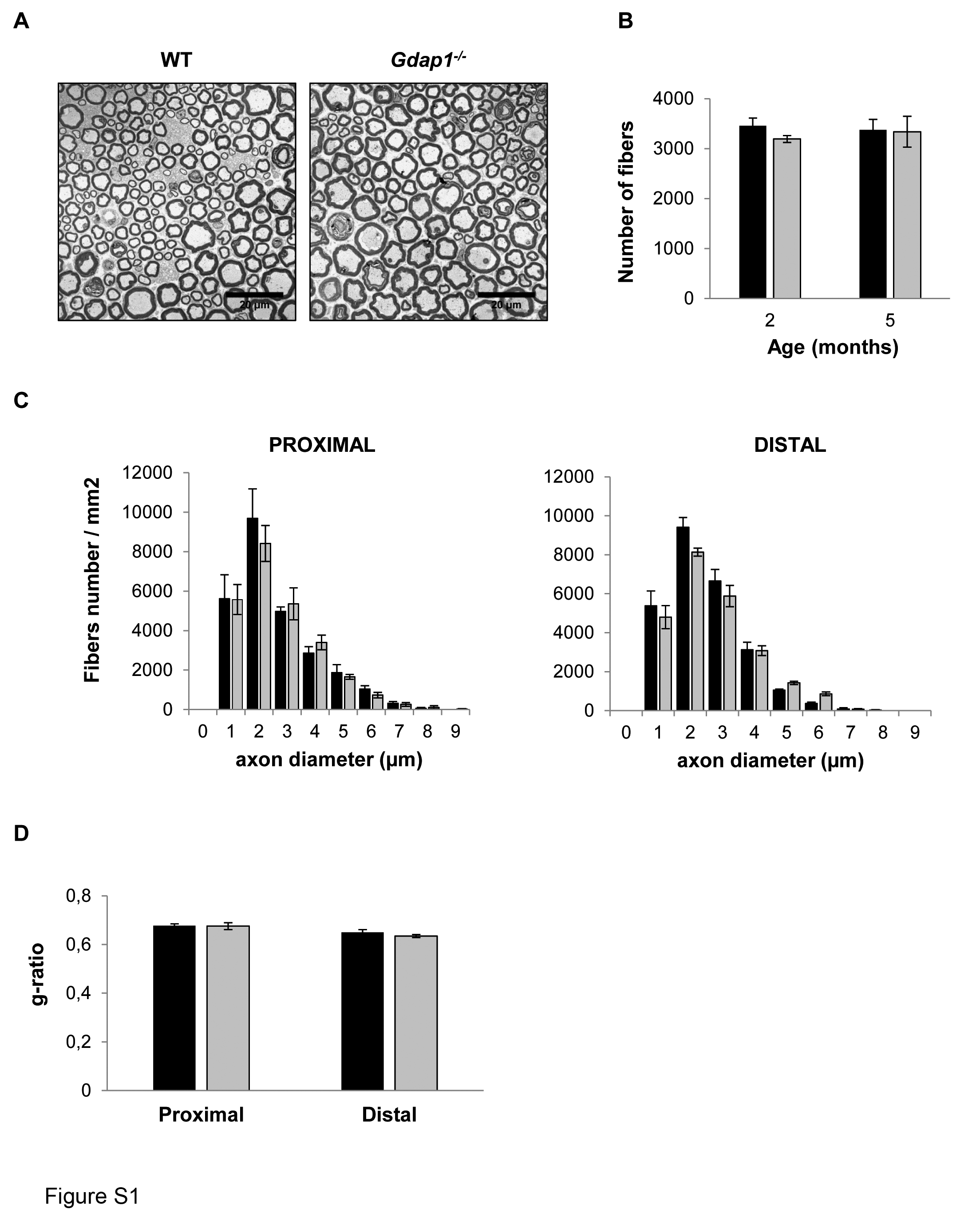

Supplement: S1 Fig — (A) Semi-thin cross sections of sciatic nerves from 5-months WT and Gdap1 -/-mice. No differences between genotypes were found analysing numbers of fibers in 2 and 5 months old mice (B). Similarly, at proximal and distal sciatic nerves axon size distribution (C) and g-ratio (D) show no differences between 5 months old control and Gdap1 -/- mice. (TIF) [file pgen.1005115.s001.tif]

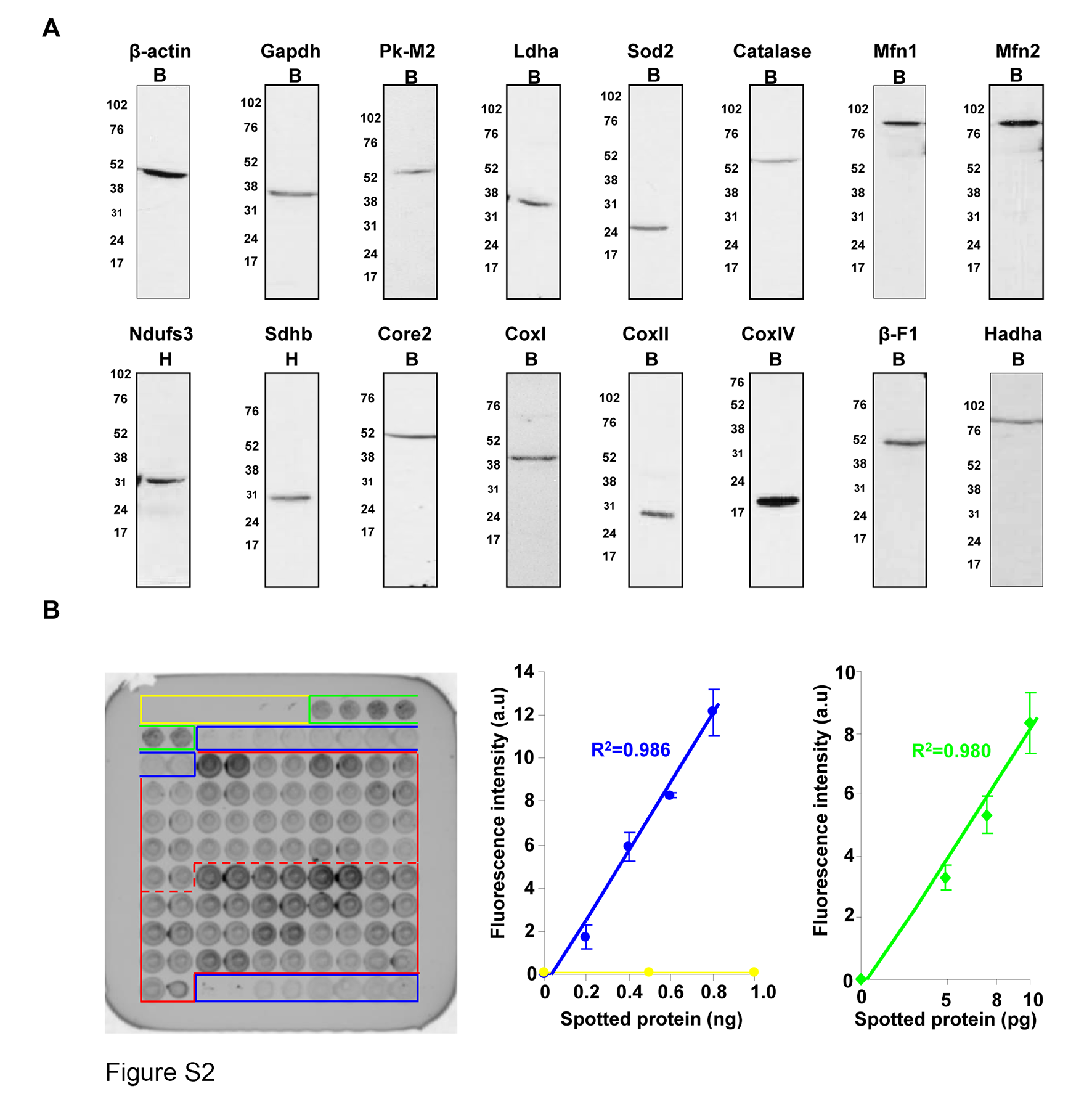

Supplement: S2 Fig — (A) 20 μg of tissue extracts (brain, B; heart, H) were fractionated on 4M urea SDS-PAGE gels, blotted against the indicated antibodies and processed for western blotting. Only antibodies that recognize a single protein band of the expected molecular mass were used in the study. The migration of molecular mass markers is indicated to the left. (B) Shows the details of the scheme of printing of RPPM. One μl samples were spotted in duplicate. Yellow boxed: negative controls of BSA; Green boxed: IgGs spotted as positive control of the secondary antibody used; Blue boxed: standard curves of HCT116 cells; Red boxed: tissue samples. The upper and lower red boxed samples correspond to different tissues. The blue plot illustrates the linear correlation that exists between the fluorescence intensity (arbitrary units, a.u.) and the amount of protein in HCT116 cell lysates. Note the absence of signal in BSA samples (yellow) and the linear increase response of the secondary antibody towards increasing content of spotted IgGs (green). Protein concentrations in the biopsies were calculated according to the fluorescence intensity obtained (TIF) [file pgen.1005115.s002.tif]

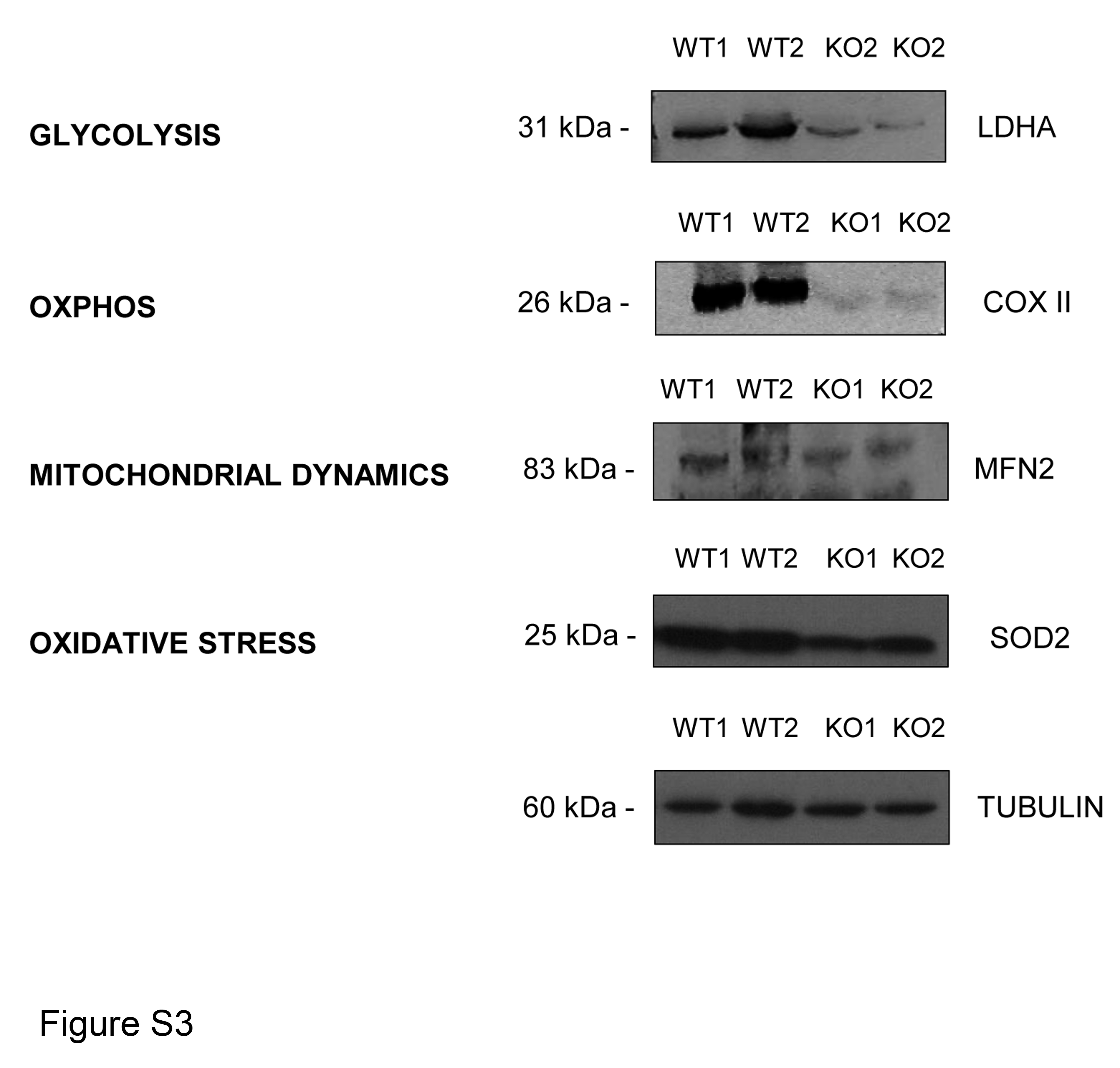

Supplement: S3 Fig — 25 μg of tissue extract from peripheral nerves (sciatic and axillar) of two wild type (WT1, WT2) and two Gdap1-KO (KO1, KO2) 5-months mice were fractionated on SDS-PAGE gels, blotted against the indicated antibodies and processed for western blotting. Note the significant reduction in the expression of LDHA, COXII and MFN2, and the presence of changes in the expression of the mitochondrial SOD2, consistent with the findings reported in RPPM (Fig 8). Representative blot of citoeskeletal tubulin as loading control. (TIF) [file pgen.1005115.s003.tif]

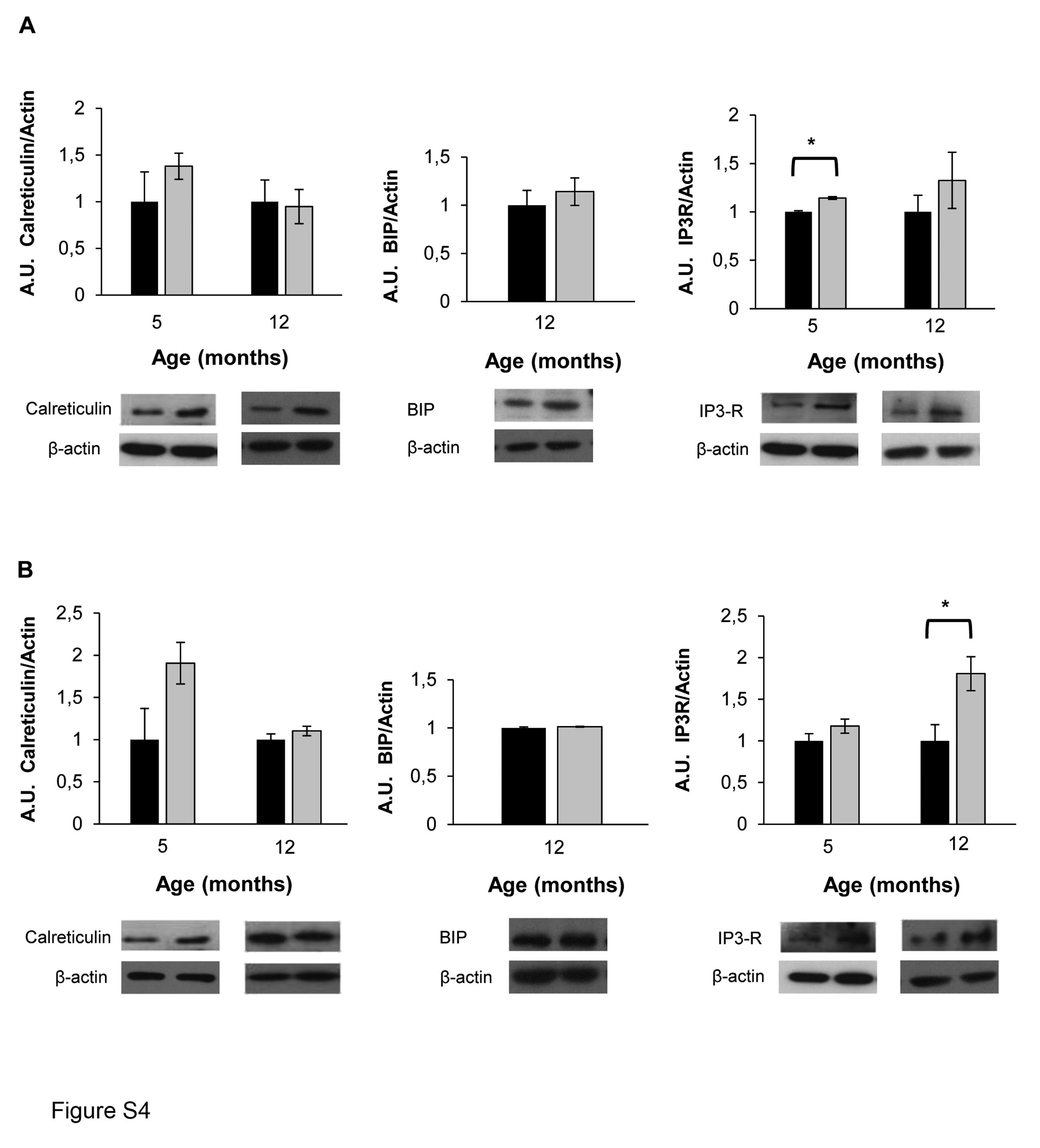

Supplement: S4 Fig — Western blot analyses of Calreticulin, BIP and IP3R in sciatic nerve (A) and lumbar spinal cord (B) biopsies of WT and Gdap1 -/-mice. Histograms show normalized intensities measured by densitometry and calculated relative to β-actin. A representative blot is shown below each graph. Error bars are SEM. p values were calculated using Student's t test (n = 6. *p<0.05). (TIF) [file pgen.1005115.s004.tif]

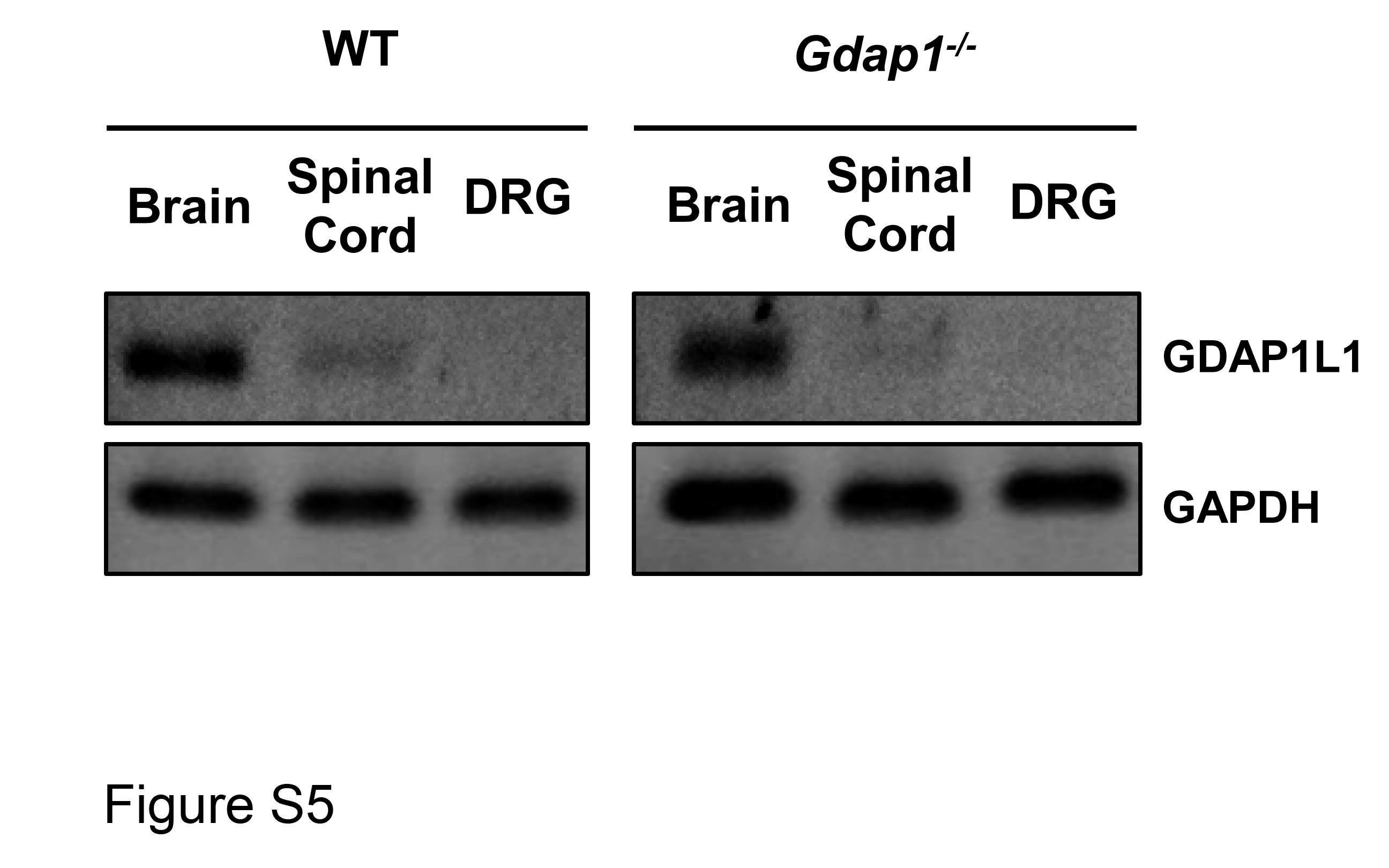

Supplement: S5 Fig — Expression pattern of Gdap1 paralogue gene Gdap1l1 by semi-quantitative reverse transcriptase PCR. No differences were observed between WT and Gdap1 -/- mice tissues. (TIF) [file pgen.1005115.s005.tif]
