# Supplementary material for: Lack of GDAP1 Induces Neuronal Calcium and Mitochondrial Defects in a Knockout Mouse Model of Charcot-Marie-Tooth Neuropathy
Source: PLoS Genet. 2015 Apr 10;11(4):e1005115. doi: 10.1371/journal.pgen.1005115 (PMC4393229; doi:10.1371/journal.pgen.1005115)
Supplement: S1 Table — (DOCX) [file pgen.1005115.s006.docx]

**S1 Table. Genetic and phenotype comparative analysis between *Gdap1* knockout mice from our study and Niemann et al.**

|  | **Present *Gdap1^-/-^* mice**  **(5 months-old)** | **Niemann’s *Gdap1^-/-^* mice**  **(19 months-old)** |
| --- | --- | --- |
| **Knock-out mouse generation** | |  |
| Exon deleted | exon 1 | exon 5 |
| Expected product | no protein | truncated protein – no protein |
| **Phenotype** | |  |
| Motor behaviour | Rota-rod defects at 3months | no phenotype (13 months) |
| NCV (m/s) | 8% reduction at 5mo | 25% reduction |
| CMAP amplitude (mV, proximal stimulation) | 42% reduction | 34% reduction |
| CMAP amplitude (mV, distal stimulation) | 39% reduction | no changes |
| **Nerve histology** |  |  |
| Axon calibres in nerve | normal in sciatic nerves | normal in plantar nerves |
| g-ratio | normal | hypomyelination |
| Number of mitochondria in axons | increased in distal and proximal sciatic nerve fibers | increased (tendency) in plantar nerve fibers |
