# Supplementary material for: Lack of GDAP1 Induces Neuronal Calcium and Mitochondrial Defects in a Knockout Mouse Model of Charcot-Marie-Tooth Neuropathy
Source: PLoS Genet. 2015 Apr 10;11(4):e1005115. doi: 10.1371/journal.pgen.1005115 (PMC4393229; doi:10.1371/journal.pgen.1005115)
Supplement: S1 Text — (DOCX) [file pgen.1005115.s007.docx]

**Supporting Information**

**Material and methods**

**Validation of the antibodies used*.*** The commercial and/or in-house produced antibodies used in RPPM were previously validated by western blotting (see Supplemental Fig. S1A). The antibodies selected only recognized a single protein band of the expected molecular mass in fractionated proteins from different mouse tissues (Supplemental Fig. S2A). Protein fractionation was carried out in 4 M urea SDS-PAGE (9 % gels). The primary monoclonal antibodies used were: anti-Ndufs3 (1:100), anti-Cox II (1:100), anti-Cox IV (1:100) and anti-Mitofusin 1 (1:1000) from Abcam; anti-SDH-B (1:500) from Invitrogen; anti-core 2 (1:1000) from Mitosciences; anti-Mitofusin 2 (1:1000) from Abnova; anti-Cox I (1:1000) from Molecular Probes; anti-catalase (1:5000) and anti-β-actin (1:10000) from Sigma-Aldrich; anti-β-F1-ATPase (1:1000), anti-GAPDH (1:1000) and anti-PK-M2 (1:1000) from [[51](#_ENREF_51)] and anti-LDHA (1:2500). Primary rabbit polyclonal antibodies used, anti-HADHA (1:100) and anti-SOD2 (1:100), were supplied by Abcam.

**Semi-quantitative reverse transcription polymerase chain reaction**. *Gdap1l1* expression was studied using reverse transcriptase polymerase chain reaction (RT-PCR). RNA from tissues was extracted using TRI Reagent (Sigma) according to the manufacturer’s instructions. One microgram of RNA was treated with Dnase I (Sigma) and transformed into cDNA using M-MLV reverse transcriptase enzyme (Sigma) and random hexamer primers. *Gdap1l1* primers from exon 2: forward, 5’-ATGCGGCTCAACCTGGGTGAGG-3’; reverse, 5’-GGCAAAGCGTCTCTGGACCCTC-3’. Cycling conditions were 95°C for 5 min, followed by 35 cycles of 95°C for 30 s and 68°C for 30 s. As a reference *Gapdh* gene was used, primers: forward, 5’-ATGGTGAAGGTCGGTGGAAC-3’; reverse 5’-GCGGAGATGATGACCCTTTTGG-3’.
